# Dual Targeting of AChE Inhibition and GPX4 Binding by Plant-Derived Compounds for the Treatment of Alzheimer’s Disease: Insights from Molecular Docking and Molecular Dynamics Simulations

**DOI:** 10.3390/pharmaceutics18070798

**Published:** 2026-06-29

**Authors:** Suheda Rumeysa Osmanlioglu Dag, Mehmet Abdullah Alagoz

**Affiliations:** 1 Department of Pharmaceutical Botany, Faculty of Pharmacy, Inonu University, Malatya 44280, Türkiye; 2 Department of Pharmaceutical Chemistry, Faculty of Pharmacy, Inonu University, Malatya 44280, Türkiye; mehmet.alagoz@inonu.edu.tr

**Keywords:** Alzheimer’s disease, AChE, ferroptosis, GPX4, in silico, molecular docking, molecular dynamics simulation

## Abstract

**Background/Objectives**: Alzheimer’s disease (AD) is primarily characterized by cholinergic dysfunction, for which acetylcholinesterase (AChE) inhibition remains the mainstay of symptomatic treatment. However, additional hypotheses such as ferroptosis—an iron-dependent form of regulated cell death—have gained prominence in explaining disease progression. Glutathione peroxidase 4 (GPX4), a critical antioxidant enzyme, plays a protective role by suppressing ferroptotic pathways. In this context, identifying phytochemicals capable of inhibiting AChE and exhibiting activator-like binding toward GPX4 may provide a dual therapeutic benefit. This study aimed to identify such dual-acting compounds through a structure-based virtual screening approach. **Methods**: A total of 3014 natural compounds were collected from three curated databases: NPACT, HIT, and HIM. Molecular docking was performed against GPX4 (7U4I) and AChE (7D9Q). Compounds demonstrating high affinity for both targets were shortlisted. Z-score normalization and statistical ranking were used to select the best two dual-target compounds. **Results**: Out of 3014 compounds, 68 showed dual-binding potential. Among these, NPACT00189 (docking scores: −6.720 kcal/mol for GPX4; −8.983 kcal/mol for AChE) and NPACT01210 (docking scores: −5.813 kcal/mol for GPX4; −9.640 kcal/mol for AChE) were identified as top candidates based on docking scores. Molecular dynamics (MD) simulations were conducted for both compounds for 250 ns on the AChE binding site and the allosteric site of GPX4. The results indicated that NPACT00189 maintained stable interactions throughout the simulation period at both targets, indicating its dual-targeting potential. **Conclusions**: NPACT00189 represents a promising dual-target candidate for further investigation in AD therapy. Its potential requires confirmation through comprehensive in vitro and in vivo studies.

## 1. Introduction

Alzheimer’s disease (AD) is a multifactorial neurodegenerative disorder characterized by progressive cognitive impairment, memory loss, and neuronal death. Despite significant research efforts, the precise etiology of AD remains elusive, with multiple intersecting pathogenic pathways contributing to its onset and progression [1,2]. Among the earliest and most consistently observed neurochemical abnormalities in AD is the dysfunction of the cholinergic system, particularly the reduction in acetylcholine (ACh) levels in the brain. Cholinesterase inhibitors (ChEIs) such as donepezil, galantamine, and rivastigmine aim to counteract this deficit by inhibiting acetylcholinesterase (AChE), thereby increasing synaptic ACh concentrations. However, these treatments offer only symptomatic relief and do not halt disease progression [3,4]. Therefore, the pursuit of new therapeutic leads is essential. These efforts may facilitate the development of effective disease-modifying agents capable of preventing or slowing disease progression. Bioactive phytochemicals have long served as a crucial resource in the drug discovery pipeline, providing candidates with limited systemic toxicity. This low-toxicity profile is critical, as it has the potential to enhance compliance during the necessary prolonged administration required for chronic conditions [5,6].

Ferroptosis, a regulated, iron-dependent form of non-apoptotic cell death characterized by the accumulation of lipid peroxides, has emerged as a potential mechanism underlying neuronal degeneration in AD [3,7]. Unlike classical necrosis or apoptosis, ferroptosis is driven by disruptions in iron homeostasis, glutathione depletion, and impaired activity of glutathione peroxidase 4 (GPX4), a key antioxidant enzyme responsible for reducing lipid hydroperoxides [8]. Several studies have shown that GPX4 plays a neuroprotective role by suppressing ferroptotic pathways. Its inhibition or downregulation sensitizes neurons to oxidative damage and ferroptotic cell death, while its activation has been associated with mitigation of AD-like pathology [9,10]. In fact, the depletion of GPX4 has been shown to result in age-dependent neurodegeneration and cognitive deficits in murine models, highlighting its central role in neuronal survival [3,7]. However, despite these promising preclinical observations, GPX4 has not yet been clinically validated as a therapeutic target for AD; therefore, GPX4 modulation should be considered an emerging and exploratory strategy rather than an established therapeutic approach.

**Figure 1:**
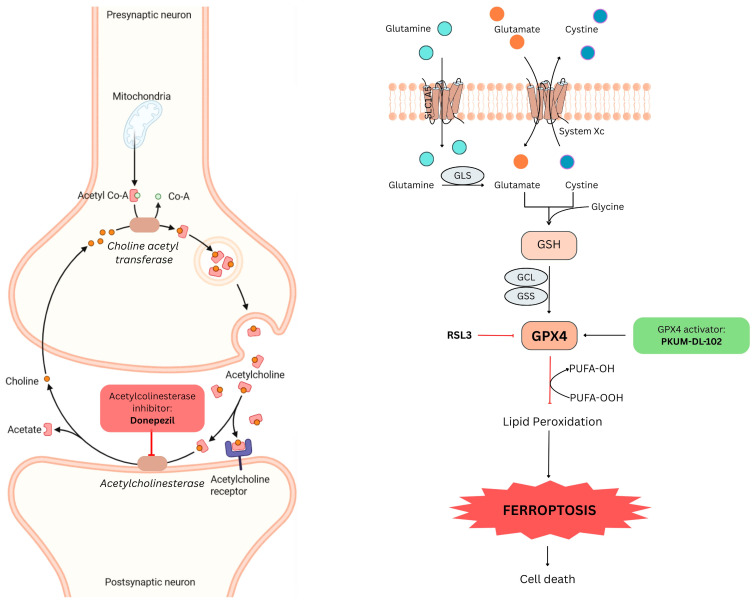
Schematic representation of cholinergic impairment and ferroptosis-related pathways in Alzheimer’s disease. Created in BioRender. Osmanlıoğlu-Dağ, Ş. R. (2026) https://BioRender.com/afvlfam.

Notably, ferroptosis and cholinergic dysfunction may converge mechanistically in AD. Iron overload in cholinergic neurons may exacerbate oxidative damage, potentiating AChE activity and disrupting neurotransmission. Furthermore, AChE itself has been implicated in the formation of Aβ fibrils, thus linking cholinergic imbalance with amyloid pathology [1,3]. The co-occurrence of these processes suggests that therapeutic agents capable of modulating ferroptosis through interactions with GPX4 regulatory regions and cholinergic signaling (via AChE inhibition) could offer dual protective effects (Figure 1).

The limitations of the traditional one-drug-single-target approach have become clear, as these agents often exhibit limited efficacy against complex diseases where pathogenesis depends on a set of interconnected biochemical events and multiple bioreceptors operating concomitantly [11]. Driven by an increased understanding of neurodegenerative disease complexity, drug development has shifted from an initial focus on single targets toward multi-target drug development, treating these diseases as networks of interconnected pathways [12]. Rationally designed multi-target drugs—also termed multimodal drugs or network therapeutics—have emerged as an attractive drug discovery paradigm. These approaches aim to enhance efficacy or improve safety relative to single-target drugs or combinations [13]. Consequently, multi-target agents have garnered significant attention as promising tools to combat challenging diseases, marking a critical new focus area in pharmacological research [11].

Medicinal plants are a source of abundant biological activities highly beneficial to human health, providing various natural remedies in the form of fruits, leaves, bark, and vegetables [14]. The diverse range of bioactive nutrients and phytochemicals present in these natural products plays a vital role in the prevention and potential management of various neurodegenerative diseases. Consequently, increasing attention is being given to these natural compounds of plant origin for their potent neuroprotective effects against oxidative damage, which may potentially hinder neurodegeneration and enhance memory and cognitive functions [15,16,17].

In this context, plant-derived natural products with multitarget properties represent promising candidates for AD drug discovery. Several bioactive phytochemicals have demonstrated both AChE inhibitory and antioxidant effects, with recent in silico studies highlighting their potential to interact favorably with GPX4 and AChE simultaneously [4,9]. Such dual-action molecules could suppress ferroptotic cell death while enhancing cholinergic neurotransmission, offering a novel strategy for disease modification.

This study aims to explore the dual modulation of GPX4 and AChE by plant-derived compounds through molecular docking and molecular dynamics simulations. These insights could inform future in vitro and in vivo investigations and ultimately contribute to the development of more effective multitarget therapies for AD.

## 2. Materials and Methods

### 2.1. Dataset Acquisition and Preprocessing

In this study, a total of 3014 plant-derived compounds were collected by downloading the Naturally occurring Plant-based Anti-cancer Compound–Activity–Target database (NPACT) (1541 compounds), Herbal Ingredients’ Targets database (HIT) (486 compounds), and Herbal Ingredients in vivo Metabolism database (HIM) (987 compounds) collections from the COCONUT (COlleCtion of Open Natural prodUcTs) database (https://coconut.naturalproducts.net/ (accessed on 1 November 2025)). These molecules were computationally evaluated against two therapeutically relevant targets GPX4 and AChE, to identify potential dual-target modulators involved in oxidative stress and neurodegeneration pathways. These collections were selected because they include curated plant-derived, herbal, and bioactive natural compounds, making them relevant chemical sources for screening potential dual-target modulators of GPX4 and AChE.

### 2.2. Molecular Docking Studies

All molecular docking studies were performed using Maestro 14.5 (Schrödinger, New York, NY, USA) with the aim of identifying dual-target compounds potentially useful against Alzheimer’s disease, acting as both Acetylcholinesterase (AChE) inhibitors and Glutathione Peroxidase 4 (GPX4) modulators. For this purpose, the crystal structures of AChE (PDB ID: 7D9Q) [18] and GPX4 (PDB ID: 7U4I) [19] were downloaded from the Protein Data Bank (www.rcsb.org) and prepared using the Protein Preparation module integrated within the Maestro software suite. Water molecules in the crystal structures were removed, hydrogen atoms were added, bond orders were adjusted, and energy minimization was performed using the OPLS4 force field. For GPX4, the allosteric pocket was selected based on previous reports describing this region as a ligand-binding site for GPX4 activator-type compounds, including PKUMDL-LC-102 [20]. Since the co-crystallized GPX4 ligand targets the catalytic site covalently, the selected allosteric pocket was justified based on previous reports describing this region as a ligand-binding site for GPX4 activator-type compounds. For AChE, the docking grid was generated around the active-site gorge, including the peripheral anionic site and catalytic/acylation region. For GPX4, docking was performed in the selected allosteric pocket located outside the catalytic site, rather than in the selenocysteine-containing catalytic center. This choice was consistent with the aim of evaluating activator-like or modulatory binding rather than catalytic-site inhibition.

A grid map of 20 Å on each side was generated for the selected binding region of each protein. Donepezil (for AChE) and PKUMDL-LC-102 (for GPX4) were selected as reference compounds. The ligands and reference compounds were prepared with the LigPrep software (Maestro 14.5) for molecular docking studies. The ligands were docked into the AChE active-site gorge and the selected GPX4 allosteric pocket 50 times at standard precision (SP) using Glide (Maestro 14.5).

To validate the docking protocol, a redocking procedure was performed using the crystal structure of AChE. The co-crystallized ligand (H1R) was removed from the binding site and subsequently redocked using the same docking parameters applied in this study. The docked pose was then superimposed with the crystallographic ligand conformation, RMSD between the two poses was calculated as 0.785 Å. This value is well below the commonly accepted threshold of 2 Å, indicating that the docking protocol can reliably reproduce the experimentally observed binding mode (Figure S1). For GPX4, docking was performed in an allosteric pocket rather than the catalytic site, as the co-crystallized ligand (L9U) represents a covalent inhibitor targeting the catalytic site.

### 2.3. Statistical Analysis and Dual-Target Ranking

To enable an objective comparison of compounds exhibiting dual affinity toward GPX4 and AChE, docking scores (X) were standardized using *Z*-score normalization, a method widely applied in inverse docking and target ranking to minimize target-specific scoring bias [21]. The initial dual-target subset consisted of 68 compounds that simultaneously met the predefined docking score thresholds for both GPX4 and AChE. *Z*-score normalization was then performed within this pre-filtered subset to rank compounds according to their relative dual-target performance.

The raw docking scores (kcal/mol) for each target were independently standardized using *Z*-score transformation. The *Z*-score (*Z*) for each raw docking score (*X*) was calculated as:

$$Z=\frac{X-\mu }{\sigma }$$

where *μ* and *σ* represent the mean and standard deviation of the docking scores, respectively (Table S2). Crucially, these parameters (*µ* and *σ*) were calculated exclusively across the pool of the 68 initial dual-target compounds for the respective protein, ensuring the *Z*-score reflects the relative affinity within the highly selective candidate subset.

Subsequently, a Composite Binding Score (*C score*) was derived for each molecule by summing the individual normalized *Z*-scores for GPX4 (*Z*_GPX4_) and AChE (*Z*_AChE_):

$$C_{score}=Z_{GPX4}+Z_{AChE}$$

This composite scoring system, a robust approach known as consensus scoring in multi-target drug design [22], provided a statistically robust framework for ranking the dual-active compounds based on their overall combined affinity, which guided the selection of the two highest-ranked candidates for subsequent molecular dynamics simulations.

### 2.4. Molecular Dynamics Simulations (MD)

The two most promising dual-target compounds identified by the molecular docking results, NPACT00189 and NPACT01210, were subjected to 250 ns MD simulations. These simulations were carried out to thoroughly investigate the stability and dynamic trajectory of the ligand–protein interactions within the GPX4 and AChE complexes. The complexes were analysed for parameters, including Root Mean Square Deviation (RMSD) and Root Mean Square Fluctuation (RMSF). The Desmond program (Maestro 14.5) was employed for all MD simulations.

The 250 ns MD simulations were conducted using the NPT ensemble for compounds NPACT00189 and NPACT01210 against AChE and GPX4. In these simulations, RMSD values of the ligands and proteins, as well as ligand–protein interactions, were evaluated. The protein-ligand complexes were immersed in a solvent by placing them in an octahedral box containing TIP3P water molecules, ensuring a minimum distance of 10 Å between the protein-ligand complexes and the box edges. The systems were rendered chemically neutral by adding Na^+^ and Cl^−^ ions, and the ionic concentration was adjusted using a 0.15 M NaCl solution. Desmond’s standard relaxation protocol was employed. The Nose–Hoover chain algorithm was used to maintain the temperature at 300 K, and the Martyna–Tobias–Klein algorithm was applied to regulate the pressure at 1.01325 bar [22,23,24].

### 2.5. MM/GBSA Calculations

The MM/GBSA method was employed to estimate the binding free energies of the protein–ligand complexes. All calculations were performed using Schrödinger’s Prime MM-GBSA module. First, single-pose MM/GBSA calculations were performed using the best docking-derived pose of each ligand–protein complex as an initial energetic estimation for compound prioritization, and these values are presented in Table 1. In addition, to provide a more rigorous energetic evaluation during MD simulations, trajectory-based MM/GBSA calculations were performed for the selected complexes. Representative structural snapshots were extracted every 5 ns from the 250 ns production MD trajectories, and MM/GBSA binding free energies were calculated for each selected snapshot. The final trajectory-based ΔGbind values were reported as mean ± standard deviation.

The binding free energy (ΔG_bind_) was calculated according to the following equation:

$$\Delta G_{bind}=\Delta E_{MM}+\Delta G_{solv}+\Delta G_{SA}$$

where ΔE_MM_ represents the molecular mechanics energy, ΔG_solv_ denotes the solvation free energy, and ΔG_SA_ corresponds to the surface area-dependent free energy contribution. All binding free energies are reported in kcal/mol [25]. Entropy was not included; therefore, the MM/GBSA values are approximate binding energy estimates.

## 3. Results

### 3.1. Molecular Docking Studies

A total of 3014 plant-based compounds were subjected to molecular docking to evaluate their binding affinities toward GPX4 and AChE. The molecular docking analysis successfully identified 68 compounds as potential dual-target ligands, demonstrating favorable binding free energies against both GPX4 (≤−3.300 kcal/mol) and AChE (≤−7.700 kcal/mol) (Tables S1 and S2). These 68 compounds were subsequently subjected to statistical ranking and MM/GBSA analysis, as described in Section 3.1.1.

#### 3.1.1. Statistical Analysis of Molecular Docking Results and MM/GBSA Calculation

A total of 68 compounds showing binding affinity toward both GPX4 and AChE were statistically evaluated to identify the most promising dual-target inhibitors. For standardization and comparative assessment, docking scores were normalized using *Z*-score transformation, independently applied to each target. This normalization allowed the generation of a composite binding score by summing the individual *Z*-scores for GPX4 and AChE, enabling a robust ranking system for dual-binding potential. It is important to note that GPX4-binding compounds are generally considered moderate binders due to the flexible allosteric site, while AChE-binding compounds are expected to exhibit stronger binding affinities owing to the well-defined active-site gorge. Among the screened compounds, NPACT00189 and NPACT01210 emerged as top-ranked candidates (Table 1). NPACT00189 and NPACT01210 have better docking scores for GPX4 compared to the reference compound, PKUMDL-LC-102. Furthermore, while the AChE docking score of NPACT00189 was similar to that of the reference inhibitor Donepezil, NPACT01210 achieved an even more favourable docking score than Donepezil.

**Figure 2:**
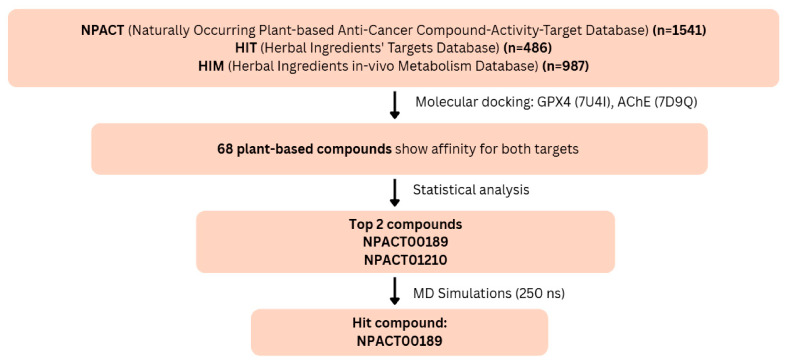
Workflow of the In Silico Screening and Selection of Dual-Target Plant-Derived Compounds Against GPX4 and AChE.

Single-pose MM/GBSA calculations were then performed using the best docking-derived conformations to obtain an initial energetic estimate for compound prioritization (Table 1). Against GPX4, the MM/GBSA binding free energies of NPACT00189 and NPACT01210 were more negative than that of PKUMDL-LC-102. For AChE, the MM/GBSA binding free energy of NPACT01210 was slightly more negative than that of donepezil. These single-pose MM/GBSA values were used only as preliminary energetic estimates during the initial selection process. Based on the docking scores, composite binding scores, and initial MM/GBSA results, NPACT00189 and NPACT01210 were selected for subsequent 250 ns MD simulations to evaluate their stability and interaction behavior within the target binding pockets (Table 2, Figure 2). To further assess the dynamic energetic stability of these complexes, trajectory-based MM/GBSA calculations were subsequently performed using representative snapshots extracted from the 250 ns MD trajectories, as described in Section 3.2.2. The reliability of the docking protocol was confirmed through a redocking validation procedure, as described in Section 2.2.

#### 3.1.2. Interactions of the Compounds at the Allosteric Site of GPX4

**Figure 3:**
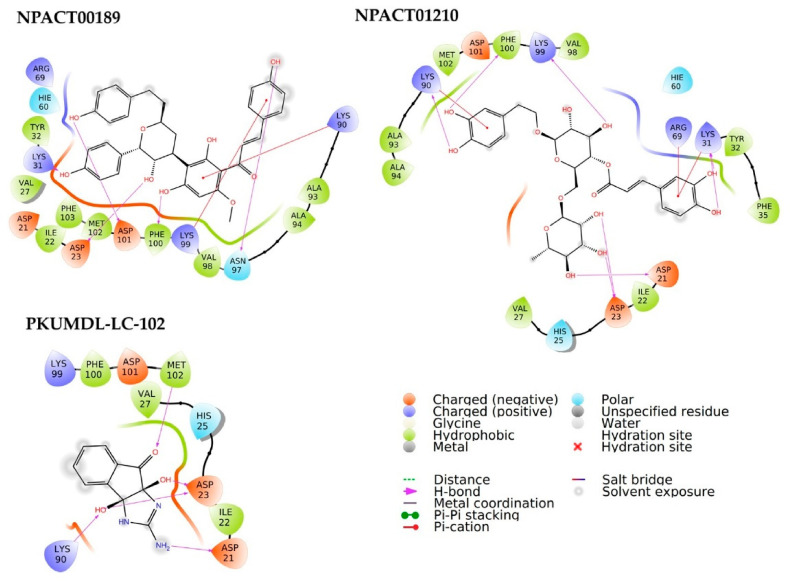
2D binding interactions of NPACT00189, NPACT01210, and PKUMDL-LC-102 within the allosteric site of GPX4. The figure illustrates the ligand–residue interactions described in Section 3.1.2 and facilitates comparison among the three complexes.

The allosteric site of GPX4 is characterized by two basic residues (Lys31, Lys90), three acidic residues (Asp21, Asp23, Asp101), and seven apolar residues (Ile22, Ala93, Ala94, Val98, Phe100, Met102, Phe103). NPACT00189, NPACT01210, and the reference PKUMDL-LC-102 exhibited similar interaction patterns within this region (Figure 3).

NPACT00189 engaged in several key interactions via its -OH functional groups, forming hydrogen bonds with the residues Asp23, Phe100, and Asp101. Additionally, its phenyl ring established a pi -cation interaction with Lys90. The ligand also showed extensive hydrophobic interactions with the surrounding apolar residues, including Ala93, Ala94, Val98, Phe100, Met102, and Phe103. Similarly, NPACT01210 formed multiple hydrogen bonds through its -OH groups with Asp21, Asp23, Lys31, Lys90, and Phe100. Hydrophobic contacts were observed with Ile22, Ala93, Ala94, Val98, Phe100, and Met102. The literature-reported GPX4 activator [20], PKUMDL-LC-102, established hydrogen bonds within the allosteric pocket with Asp21, Asp23, Lys90, and Met102. Hydrophobic interactions were noted with Ile22, Val98, Phe100, Met102, and Phe103.

Collectively, the analysis indicates that both NPACT00189 and NPACT01210 interact with the key basic (Lys90), acidic (Asp23, Asp101), and apolar residues that characterize the GPX4 allosteric binding region, exhibiting an interaction profile similar to the known activator PKUMDL-LC-102.

#### 3.1.3. Interactions of the Compounds at the Active Site of AChE

**Figure 4:**
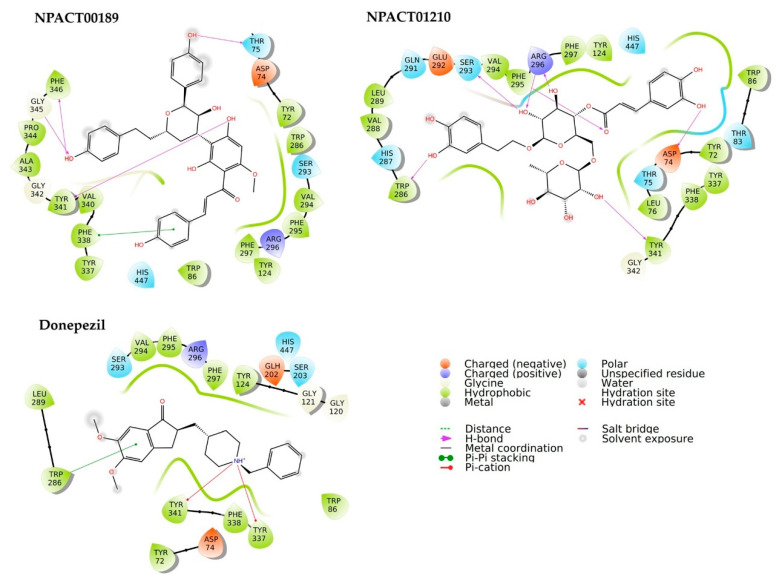
2D binding interactions of NPACT00189, NPACT01210, and Donepezil within the active site of AChE. The figure illustrates the ligand–residue interactions described in Section 3.1.3 and facilitates comparison among the three complexes.

The deep and narrow active-site gorge of AChE comprises two major ligand-binding regions: the peripheral anionic site (PAS) and the acylation or catalytic site (AS). The PAS, located near the entrance of the gorge, features the key aromatic residue Trp286, while the AS contains the catalytic triad—Ser203, Glu334, and His447—as well as the critical aromatic residue Trp86 positioned near the base of the gorge (Figure 4).

NPACT00189 primarily interacted with apolar residues within the active-site region, including Tyr72, Leu76, Trp86, Tyr124, Val294, Trp286, Phe295, Phe297, Tyr337, Val340, Ala343, and Pro344 via hydrophobic interactions. It also established a pi–pi stacking interaction with Phe338 and formed hydrogen bonds with Tyr75, Tyr341, Gly345, and Phe346. In its initial binding pose, NPACT00189 showed limited interaction with the key residues of the PAS and AS; however, MD simulations indicated that the ligand migrated closer to the active site over time, increasing its interaction with crucial catalytic residues (Figure 4).

NPACT01210 formed hydrogen bonds with several residues in the catalytic site, including Asp74, Trp286, Ser293, Phe295, Arg296, and Tyr341. Additionally, the ligand engaged in hydrophobic interactions with Tyr72, Leu76, Trp86, Tyr124, Val288, Leu289, Val294, Tyr337, and Phe338 (Figure 4). As a positive control, donepezil interacted with Trp286 via pi–pi stacking and formed pi–cation interactions with Tyr341 and Tyr337, in addition to hydrophobic interactions with residues Tyr72, Trp86, Tyr124, Trp286, Leu289, Val294, Phe295, Phe297, and Phe338 (Figure 4).

### 3.2. Comparative MD Analysis of GPX4 and AChE Complexes

According to the molecular docking results, NPACT00189 and NPACT01210 showed favorable binding scores against both GPX4 and AChE. Based on these results, 250 ns MD simulations were performed for NPACT00189, NPACT01210, PKUMDL-LC-102, and donepezil to comparatively evaluate ligand stability, protein backbone stability, and ligand–protein interaction persistence within the GPX4 allosteric pocket and the AChE active-site gorge.

#### 3.2.1. Dynamic Stability and Interaction Persistence Based on 250 ns MD Simulations

**Figure 5:**
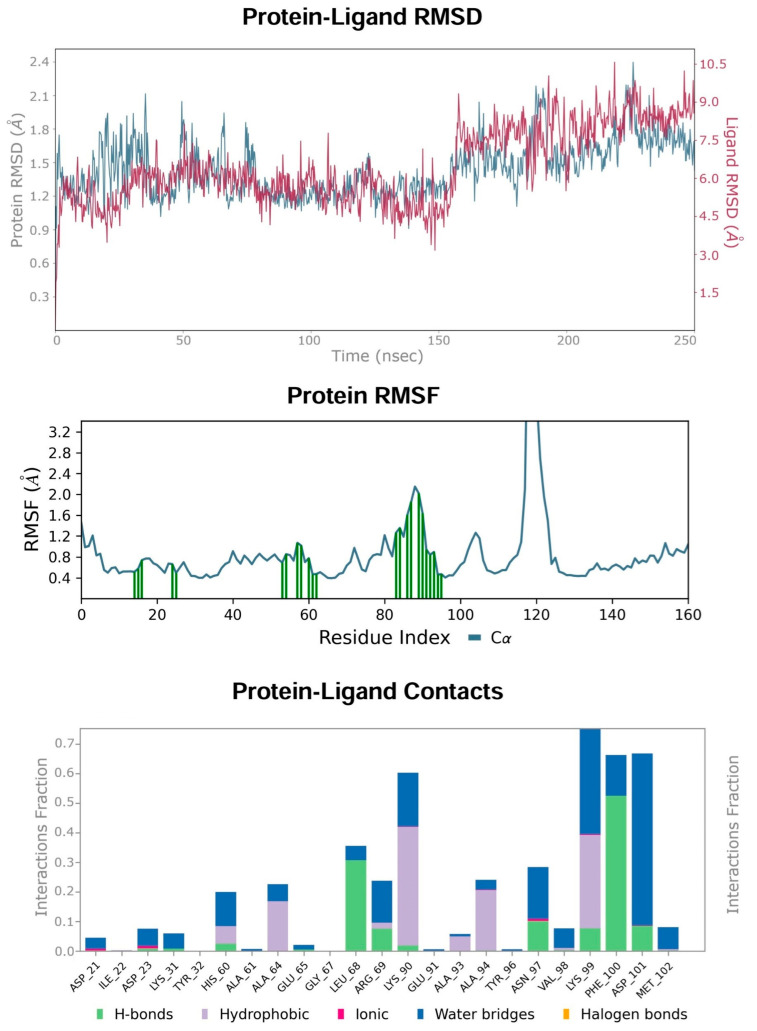
250 ns MD simulation analysis of NPACT00189 within the allosteric site of GPX4, illustrating the protein–ligand RMSD, protein RMSF, and protein–ligand contact profiles. In the RMSD plot, the blue trace represents protein Cα RMSD, whereas the red trace represents ligand RMSD. The vertical green lines in the RMSF plot indicate residues contacted by the ligand during the simulation.

Among the GPX4 complexes, NPACT00189 showed the most persistent interaction profile within the allosteric site. The ligand exhibited RMSD fluctuations between 3.5 Å and 7.5 Å during the initial 0–150 ns followed by increased fluctuations ranging from 6.0 Å to 9.5 Å after 150 ns. The average RMSD value between 160 and 250 ns after the ligand became stable was calculated to be approximately 3.3 Å. The Cα RMSD of GPX4 remained relatively stable, fluctuating between 0.9 Å and 2.2 Å throughout the simulation, indicating a well-maintained protein structure. The RMSF analysis revealed minor fluctuations (<3.2 Å) primarily in the 110–125 residue region, with all other fluctuations remaining below 2.4 Å (RMSF < 2.4 Å) (Figure 5). NPACT00189 maintained persistent interactions with key residues, including Lys90, Phe100, and Asp101, primarily through hydrogen bonding and water-mediated contacts. Approximately 99% of the simulation time showed consistent interactions with Lys99, contributing to ligand stability (Figure S2). Up to 150 ns, the compound sustained interactions with allosteric site residues, especially the acidic residue Asp101; the basic residue Lys90; and hydrophobic residues Ala94, Phe100, and Met102. However, the compound showed limited interaction with acidic residues Asp21 and Asp23. Following this initial 150 ns period, a conformational rearrangement occurred, leading to a reduction in interactions with Asp21, Asp23, Lys90, and Met102, and the emergence of new interactions with Lys31, Ala64, Leu68, Arg69, and Val98. Throughout the simulation, the ligand maintained critical contacts with Asp101 and Phe100, showing that interactions with Lys90, Phe100, and Asp101 may play a key role in anchoring the molecule within the GPX4 allosteric site (Figure 5).

**Figure 6:**
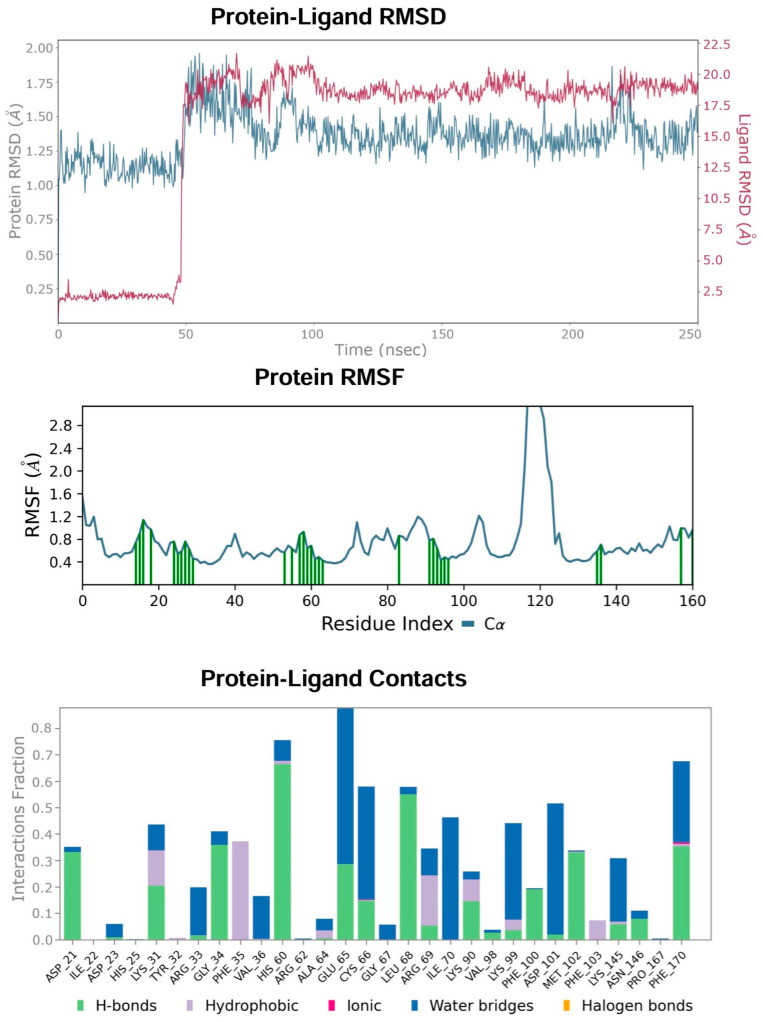
250 ns MD simulation analysis of NPACT01210 within the allosteric site of GPX4, illustrating the protein–ligand RMSD, protein RMSF, and protein–ligand contact profiles. In the RMSD plot, the blue trace represents protein Cα RMSD, whereas the red trace represents ligand RMSD. The vertical green lines in the RMSF plot indicate residues contacted by the ligand during the simulation.

In contrast to NPACT00189, NPACT01210 showed less persistent residence within the GPX4 allosteric pocket. NPACT01210 initially exhibited stable binding within the GPX4 allosteric site during the first 47 ns of the MD simulation, maintaining an average ligand RMSD of approximately 1.0 Å. However, following this period, a significant conformational shift was observed, with the ligand RMSD increasing and fluctuating between 17.5 Å and 21.5 Å, indicating displacement from the original binding site. Throughout the entire simulation, the RMSD of the protein’s Cα atoms remained within the range of 1.0 Å to 2.0 Å, reflecting the overall structural integrity of the protein. RMSF analysis demonstrated minimal residue fluctuations, with all values below 3.0 Å (RMSF < 3.0 Å), suggesting a relatively stable protein backbone (Figure 6). The compound interacted with Glu65 (99%), His60 (80%), and Phe170 (75%) by forming hydrogen bonds and a water bridge during a significant portion of the simulation (Figure S3). From 0 to 47 ns of the simulation, it interacted with residues located in the allosteric region, including Asp21, Asp23, Lys31, Lys90, Lys99, Phe100, Met102, and Phe103. However, due to a significant conformational change, the ligand’s interactions with residues such as Asp21, Asp23, Lys31, Lys90, Val98, Phe100, Met102, and Phe103 were disrupted. After 47th ns, the ligand moved away from the allosteric site and began to interact with Glu34, Val36, Ala64, Glu65, Cys66, Leu68, Ile70, Lys145, and Phe170. Although the ligand remained stable from 50 ns onwards, it was observed to move away from the allosteric pocket (Figure S3). MD simulation results for the NPACT01210–GPX4 complex indicated that the ligand dissociated from the allosteric binding site after 47 ns, accompanied by a marked reduction in interactions with key residues. These observations suggest that NPACT01210 may experience displacement from the original allosteric binding pocket during the simulation. Given the flexible nature of the GPX4 allosteric region, such behavior may reflect dynamic pocket rearrangements rather than simple ligand instability. Although molecular docking initially suggested NPACT01210 as a candidate compound targeting both GPX4 and AChE, the MD analyses indicate that its residence within the GPX4 allosteric region is less persistent under the simulated conditions.

**Figure 7:**
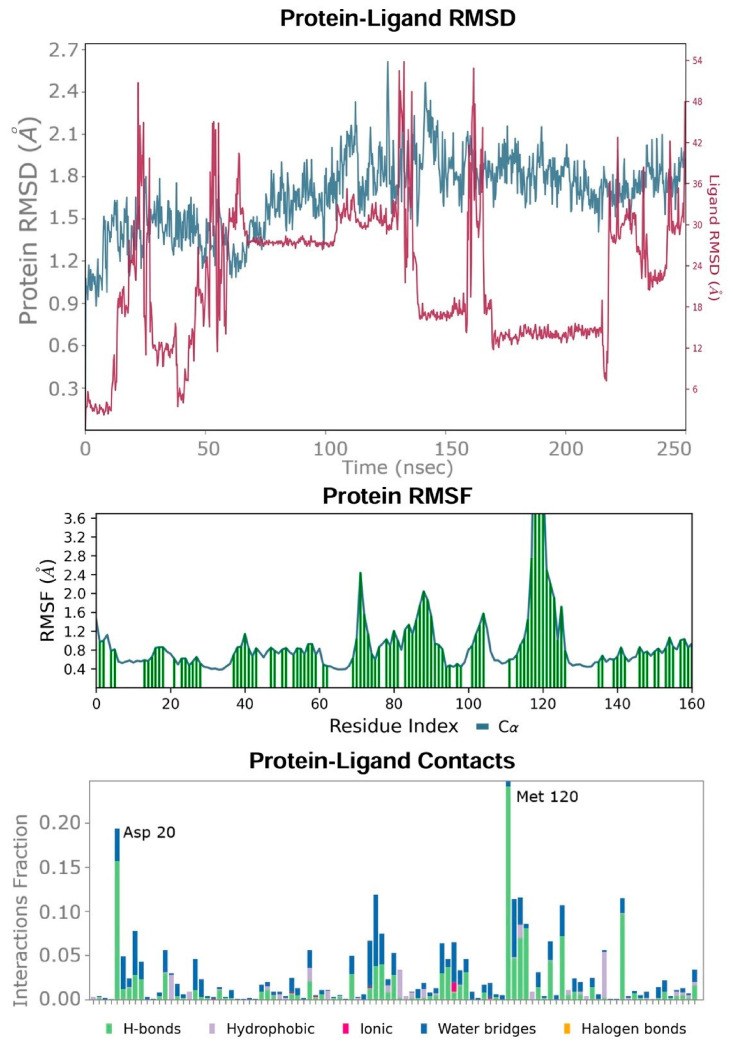
250 ns MD simulation analysis of PKUMDL-LC-102 within the allosteric site of GPX4, illustrating the protein–ligand RMSD, protein RMSF, and protein–ligand contact. In the RMSD plot, the blue trace represents protein Cα RMSD, whereas the red trace represents ligand RMSD. The vertical green lines in the RMSF plot indicate residues contacted by the ligand during the simulation.

The compound PKUMDL-LC-102, known as a GPX4 activator-type was used as a reference molecule for comparison within the GPX4 allosteric pocket. However, PKUMDL-LC-102 showed unstable ligand behavior during the MD simulation. Examination of the protein–ligand RMSD plot shows marked fluctuations in the ligand’s RMSD values (Figure 7). Although the compound remains relatively stable during certain segments of the simulation (specifically between 60–110 ns and 160–215 ns), it does not display an overall stable binding profile. Therefore, PKUMDL-LC-102 was used mainly as a literature-based reference for comparison and workflow support, rather than as evidence of stable GPX4 binding. The RMSD values of the protein Cα atoms range between 0.9 and 2.4 Å, which is within an acceptable range. In the protein RMSF analysis, fluctuations remain below 3.6 Å (RMSF < 3.6 Å). Throughout the simulation, the compound interacts with multiple residues; however, during significant portions of the trajectory, it predominantly forms hydrophobic contacts and water-bridge interactions with Lys20 and Met120 (Figure S4).

**Figure 8:**
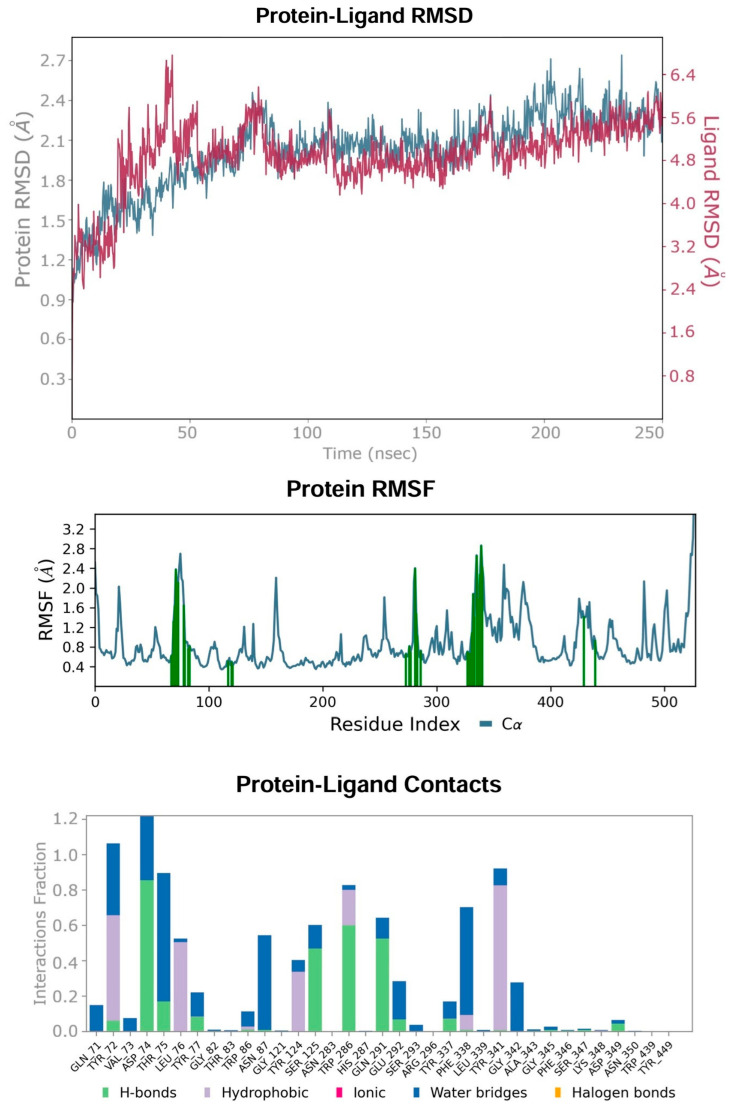
250 ns MD simulation analysis of NPACT00189 within the active gorge of AChE, illustrating the protein–ligand RMSD, protein RMSF, and protein–ligand contact profiles. In the RMSD plot, the blue trace represents protein Cα RMSD, whereas the red trace represents ligand RMSD. The vertical green lines in the RMSF plot indicate residues contacted by the ligand during the simulation.

The MD simulation of the NPACT00189–AChE complex throughout the 250 ns revealed an average ligand RMSD of approximately 3.5 Å, while the RMSD of the protein Cα atoms remained stable around 1.7 Å. The RMSF profile of the protein showed minimal fluctuations, all remaining below 2.8 Å (RMSF < 2.8 Å), indicating overall structural stability (Figure 8). Throughout the simulation, NPACT00189 maintained persistent interactions—hydrogen bonding, hydrophobic contacts, and water bridges—with the key residue Trp286 for approximately 70% of the total simulation time. Additionally, the ligand engaged consistently with residues located near the base of the peripheral anionic site (PAS), including Asp74 (hydrogen bond, water bridge), Tyr124 (hydrophobic, water bridge), Ser125 (hydrogen bond, water bridge), and Tyr341 (hydrophobic, water bridge) (Figure 8). Continuous interactions with Trp286, Asp74, Thr75, Tyr124, and Tyr341 throughout the trajectory further support the notion that NPACT00189 remained stably accommodated within the AChE active-site gorge (Figure S5). These findings suggest a robust and sustained binding affinity of NPACT00189 to the AChE binding cavity during the MD simulation.

**Figure 9:**
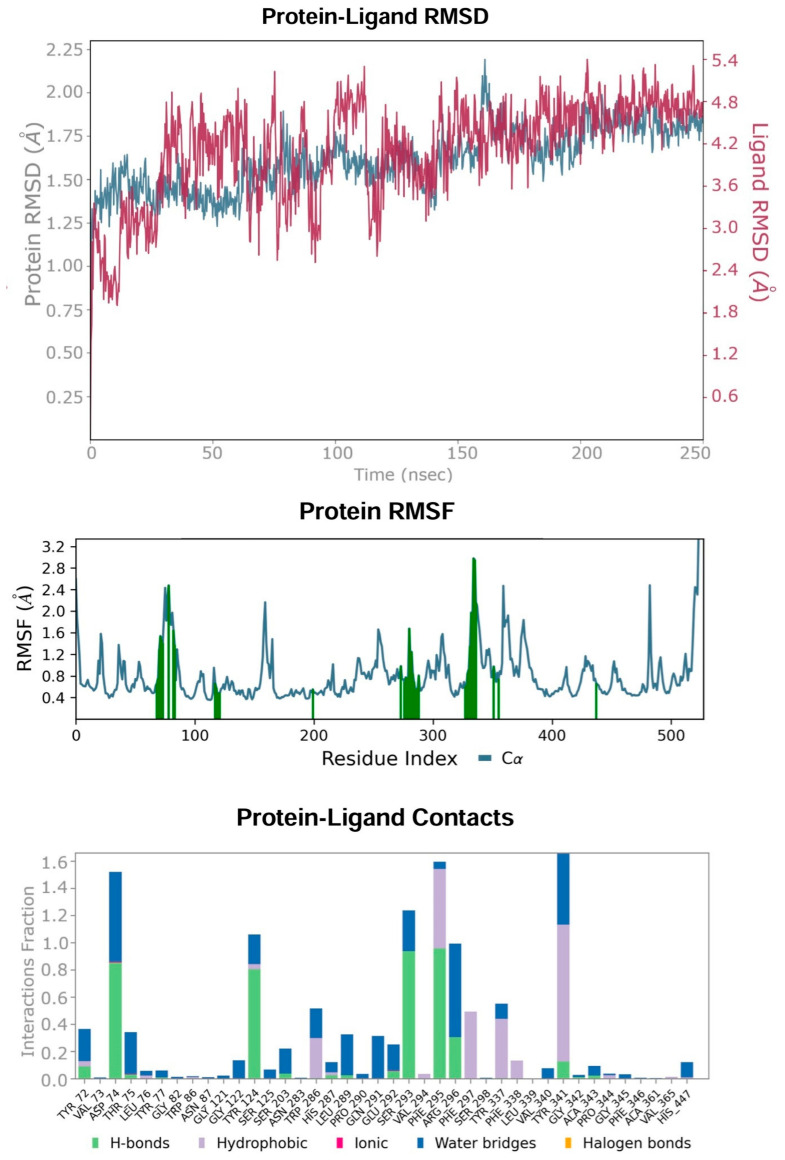
250 ns MD simulation analysis of NPACT01210 within the active gorge of AChE, illustrating the protein–ligand RMSD, protein RMSF, and protein–ligand contact profiles. In the RMSD plot, the blue trace represents protein Cα RMSD, whereas the red trace represents ligand RMSD. The vertical green lines in the RMSF plot indicate residues contacted by the ligand during the simulation.

Throughout the 250 ns MD simulation, the average RMSD value of NPACT01210 within the AChE binding pocket was approximately 3.0 Å, while the average Cα RMSD of the protein remained around 0.75 Å, indicating overall structural stability (Figure 9). During a significant portion of the simulation, the ligand formed hydrogen bonds and water bridges with residues such as Asp74 (93%), Tyr124 (68%), and Phe293 (75%). Persistent hydrophobic interactions with Phe295 and Tyr341 were also detected, lasting for more than 95% of the simulation (Figure 9). In addition, stable contacts were observed with Ser293, Arg296, and Tyr337 (Figure S6). These findings indicate that NPACT01210 remained structurally stable within the AChE binding site. However, its limited interactions with key residues in the peripheral anionic site (PAS) and acylation site (AS) reduce its potential to act as an effective AChE inhibitor.

**Figure 10:**
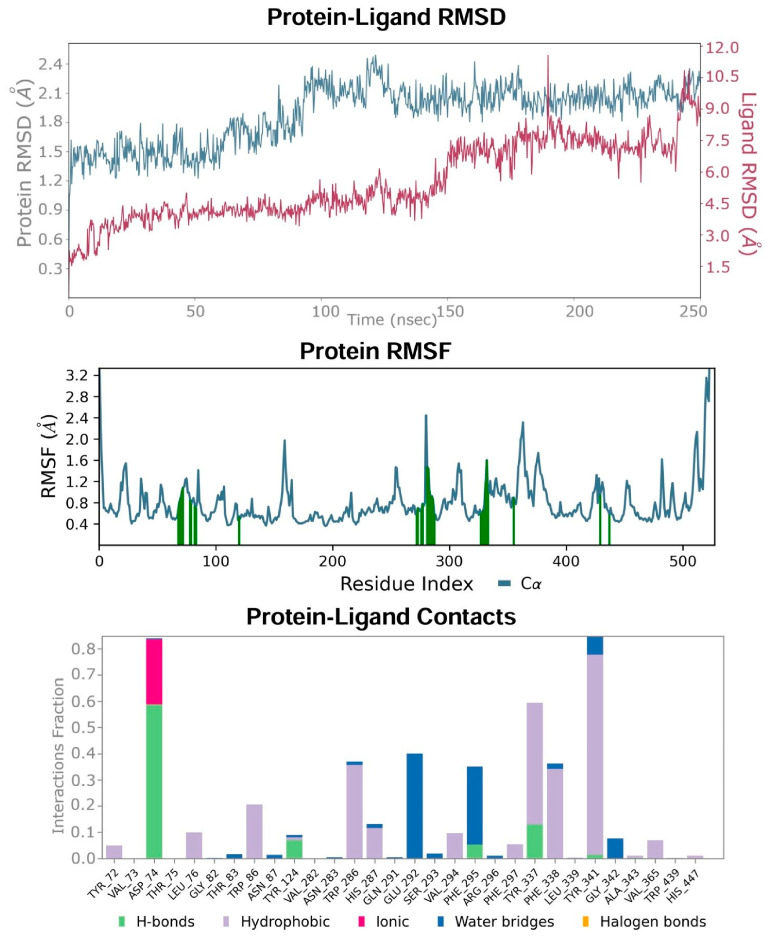
250 ns MD simulation analysis of Donepezil within the active gorge of AChE, illustrating the protein–ligand RMSD, protein RMSF, and protein–ligand contact. In the RMSD plot, the blue trace represents protein Cα RMSD, whereas the red trace represents ligand RMSD. The vertical green lines in the RMSF plot indicate residues contacted by the ligand during the simulation.

Finally, donepezil was evaluated as the reference inhibitor for comparison within the AChE active-site gorge. During the initial phase of the simulation (0–140 ns), the ligand exhibited RMSD values ranging from 1.5 to 4.5 Å. In the later phase (160–245 ns), the RMSD increased and fluctuated between 6 and 9 Å, indicating periods of reduced conformational stability. Throughout the simulation, the RMSD of the protein Cα atoms remained consistently within the 0.9–2.4 Å interval, and the protein RMSF values were below 2.8 Å (RMSF < 2.8 Å), reflecting a stable protein backbone (Figure 10). Donepezil interacted predominantly with Asp74 (ionic interactions and hydrogen bonding), Trp341 (hydrophobic contacts and water-bridge interactions), and Tyr337 (hydrophobic and water-bridge interactions). Additionally, the ligand maintained intermittent interactions with Asp74, Tyr337, Phe338, and Tyr341 across various segments of the simulation trajectory (Figure S7), suggesting that these residues contribute to the stabilization of the ligand within the AChE active-site gorge. For the validation of the molecular docking protocol, the donepezil molecule present in the crystal structure was removed, energy-minimized, and subsequently redocked. The resulting RMSD between the crystallographic and redocked poses was calculated as 0.9008 Å.

In addition to the protein–ligand contact analyses, the time-dependent evolution of hydrogen bonds was evaluated throughout the 250 ns MD trajectories for the selected ligand–protein complexes. The H-bond evolution profiles showed dynamic but sustained hydrogen-bond formation during the simulations, supporting the temporal persistence and fluctuation of ligand–protein interactions observed in the contact analyses. These profiles were provided in the Supplementary Materials for NPACT00189–AChE, NPACT00189–GPX4, NPACT01210–AChE, and NPACT01210–GPX4 complexes (Figure S8).

#### 3.2.2. Trajectory-Based MM/GBSA Binding Free Energy Analysis

Trajectory-based MM/GBSA analysis was performed to further evaluate the energetic stability of the selected ligand–protein complexes during the 250 ns MD simulations. The mean ± standard deviation values calculated from representative trajectory snapshots are summarized in Table 3.

**Figure 11:**
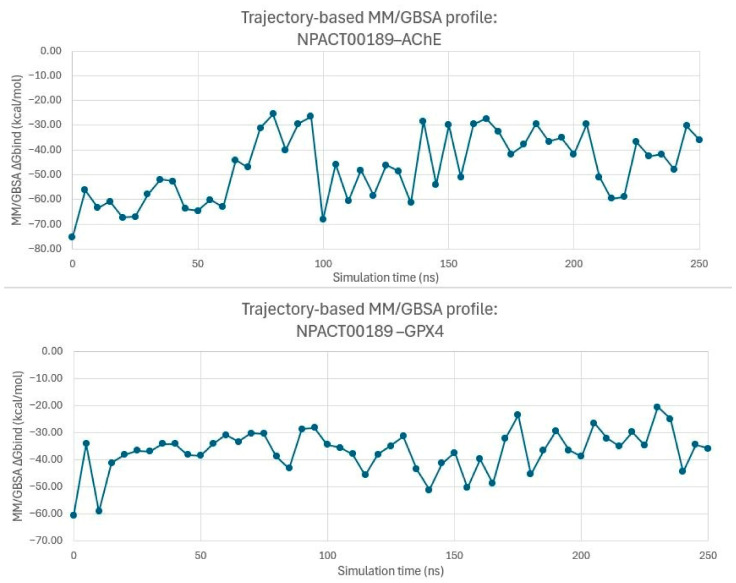
Trajectory-based MM/GBSA binding free energy profiles of the final hit compound NPACT00189.

For the final hit compound NPACT00189, trajectory-based MM/GBSA profiles were included in the main manuscript for both the AChE and GPX4 complexes (Figure 11). NPACT00189 showed favorable average binding energies throughout the MD trajectories, with ΔGbind values of −46.97 ± 13.65 kcal/mol for AChE and −36.92 ± 7.96 kcal/mol for GPX4 (Table 3). Although moderate fluctuations were observed, particularly in the AChE complex, the binding energies remained generally favorable over the simulation period, supporting the sustained energetic interaction of NPACT00189 with both targets.

The corresponding trajectory-based MM/GBSA results for NPACT01210 were also summarized in Table 3 and provided as a supplementary figure to support transparency and comparative interpretation (Figure S9). For NPACT01210, the mean ΔGbind values were −73.90 ± 10.28 kcal/mol for AChE and −37.83 ± 9.76 kcal/mol for GPX4 (Table 3). Although NPACT01210 showed favorable average MM/GBSA binding energies, the overall MD-based structural stability and interaction persistence analyses supported the selection of NPACT00189 as the final hit compound.

### 3.3. Binding Pocket Complementarity Analysis

**Figure 12:**
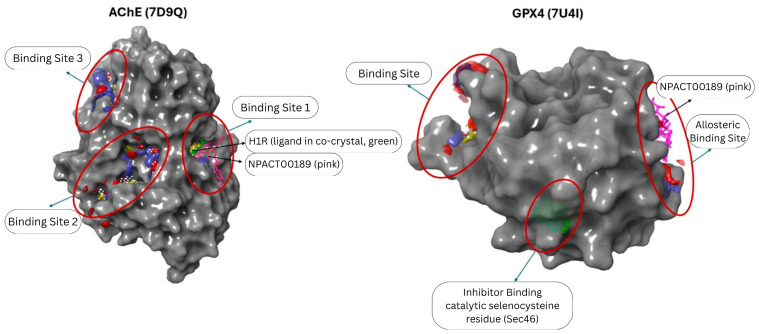
Structural comparison of the binding pockets of AChE (7D9Q) and GPX4 (7U4I) based on SiteMap analysis.

To establish the structural basis for dual-target engagement, SiteMap analysis was performed for both proteins using the SiteMap module implemented in Schrödinger Maestro (Table 4 and Table S3). SiteMap analysis was used to characterize the selected docking pockets and to support their structural complementarity with the investigated ligands. Only the selected Site 1 pockets, which correspond to the docking regions used in this study, are reported in Table 4 and Table S3. For AChE, six potential binding sites were identified. Among these, Site 1 exhibited the highest SiteScore and Dscore values (1.147 and 1.180, respectively) and corresponded to the binding region of the co-crystallized ligand H1R. For GPX4, two potential binding sites were identified, one of them was allosteric pocket. The SiteScore values of these two pockets were comparable, suggesting that both regions possess favorable physicochemical characteristics for ligand binding (Figure 12). In addition, the canonical GPX4 inhibitor-binding region centered around the catalytic selenocysteine residue (Sec46), where many inhibitors form covalent adducts with the enzyme, is also illustrated in Figure 12. Despite the volumetric difference between the AChE active-site gorge (454 Å^3^) and the GPX4 allosteric pocket (92 Å^3^), several shared physicochemical features facilitate dual target ligand recognition: First, both pockets present hydrophobic clusters formed by aromatic residues. AChE contains an aromatic cage (Trp86, Phe295, Tyr341, Trp439) that engages planar ligand scaffolds through π-π stacking, while GPX4 provides a compact hydrophobic region (Phe100, Met102) suitable for lipophilic substituents. Second, the donor/acceptor balance is remarkably similar (AChE: 0.819; GPX4: 0.991), indicating both pockets accommodate ligands with balanced hydrogen-bond pharmacophores. The philic scores (0.853 vs. 0.629) confirm sufficient polar character in both sites for electrostatic interactions. Third, both pockets utilize aspartate residues for electrostatic anchoring—Asp74 in AChE and the Asp21/Asp23 dyad in GPX4. Collectively, these similarities provide a structural rationale supporting the potential of certain ligands to interact with both targets beyond binding affinity considerations.

### 3.4. In Silico ADMET Analyses

To evaluate the pharmacokinetic suitability of the identified lead compound (NPACT00189), in silico ADMET predictions were performed using PreADMET web server, SwissADME web server, and QikProp tools (Maestro 14.5). PreADMET analysis predicted favorable BBB penetration with a logBB value of 0.814, suggesting potential brain exposure. In contrast, SwissADME BOILED-Egg classification predicted that the compound may not readily cross the blood–brain barrier through passive diffusion, likely due to its relatively high molecular weight (598.64 g/mol) and elevated topological polar surface area (156.91 Å^2^). QikProp analysis further indicated limited passive CNS permeability (QPlogBB = −2.845) suggesting restricted passive diffusion across the BBB. However, the compound exhibited moderate lipophilicity (consensus LogP = 4.25) and acceptable predicted oral absorption (~58%), which may still support systemic exposure. Taken together, these tool-dependent results suggest that NPACT00189 may exhibit limited passive BBB permeability, although brain exposure could still occur through alternative mechanisms such as transporter-mediated uptake or optimized drug delivery strategies. The predicted toxicity profile of NPACT00189 was evaluated using the DataWarrior 06.01.00 software. Several toxicity endpoints, including mutagenic, tumorigenic, reproductive, and irritant effects, were assessed. The analysis did not indicate any predicted cytotoxic or high-risk toxicological properties for the compound. The differences among BBB permeability predictions likely reflect the use of different algorithms, descriptors, and training datasets by each computational tool.

### 3.5. Complementary In Silico Target Prediction Analysis

**Figure 13:**
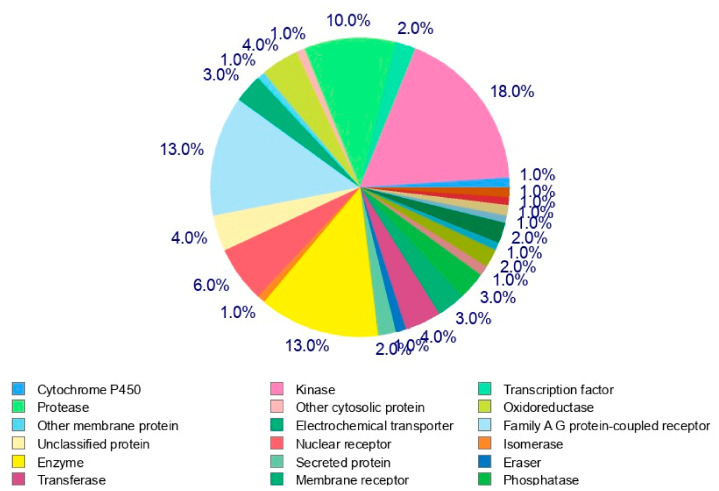
SwissTargetPrediction target class distribution for NPACT00189.

To further evaluate the potential biological targets of the lead compound, an in silico target prediction analysis was performed using the SwissTargetPrediction server with *Homo sapiens* selected as the target organism. The predicted target distribution indicated enrichment primarily in enzyme-related classes, including kinases, oxidoreductases, and other catalytic proteins. Notably, oxidoreductase-associated targets were represented among the predicted classes, supporting the plausibility of interactions with redox-regulating enzymes such as GPX4. The overall distribution of predicted targets is presented in Figure 13.

## 4. Discussion

Recent investigations have established the pivotal involvement of ferroptosis in neuronal demise across various neurological disorders [26]. Key attributes of ferroptosis, such as iron dysregulation and reactive oxygen species (ROS) accumulation, are directly relevant to Alzheimer’s disease (AD) pathology [27]. This evidence suggests that ferroptosis may contribute to AD pathology and neuronal vulnerability [28]. In parallel, the cholinergic system remains a major therapeutic axis in AD, while oxidative stress is also closely associated with AD pathophysiology [29,30,31]. Previous virtual screening and MD-based studies have supported the utility of computational approaches for identifying AChE-directed candidates, while ferroptosis-related mechanisms, including GPX4 dysregulation, have also been increasingly discussed in AD research [32,33].

A unique biochemical feature of GPX4 is the presence of a catalytic selenocysteine residue (Sec46), which distinguishes this enzyme from most other antioxidant proteins. The selenium-dependent catalytic activity is essential for preventing uncontrolled lipid peroxidation and ferroptotic cell death. Loss or inactivation of the selenocysteine-containing GPX4 enzyme has been shown to trigger rapid ferroptosis in neuronal and non-neuronal cells [34]. From this perspective, the selected GPX4 allosteric pocket is biologically relevant because it corresponds to the ligand-binding region previously described for GPX4 activator-type compounds, including PKUMDL-LC-102. This pocket contains charged and hydrophobic residues that may contribute to ligand-induced conformational stabilization of GPX4 [35]. Therefore, ligand binding at this region may potentially influence GPX4-related ferroptosis regulation. However, in the present study, this effect should be interpreted as putative allosteric modulation rather than direct evidence of enzymatic activation.

Accordingly, modulation of GPX4 may represent a potential exploratory strategy for regulating ferroptosis in neurodegenerative disorders such as Alzheimer’s disease. In this context, targeting regulatory regions of GPX4 rather than directly interfering with the catalytic selenocysteine site may provide a strategy to influence enzyme dynamics without inducing complete enzymatic inhibition. In the present study, the screened compounds were docked into a predicted allosteric pocket of GPX4, located outside the catalytic center. Interaction with such regulatory regions may alter local conformational dynamics and potentially support the stabilization or modulation of GPX4 activity while avoiding disruption of the essential selenium-dependent catalytic machinery. Similar regulatory targeting strategies have been proposed for modulating ferroptosis-related pathways in neurodegenerative disorders [34].

The simultaneous modulation of GPX4 and AChE represents a promising polypharmacological strategy for neurodegenerative diseases, particularly AD, where oxidative stress and cholinergic dysfunction are both prominent pathological features [30,31]. Therefore, targeting both the allosteric site of GPX4 and the AChE active site may provide a mechanistically relevant strategy for AD-directed drug discovery. This concept is also consistent with the broader literature on natural multi-target compounds in AD research. Several plant-derived compounds, particularly polyphenols and flavonoid-like scaffolds, have been investigated because they may combine AChE inhibitory, antioxidant, anti-inflammatory, and ferroptosis-modulating effects. For example, the natural polyphenol tannic acid has been reported to mitigate amyloid-β-induced ferroptosis in AD-related models through a dual-acting mechanism involving GPX4 enhancement and ferroptosis regulation [9].

Based on subsequent molecular docking and statistical ranking analysis, NPACT00189 and NPACT01210 emerged as the most promising dual-target modulators. Both compounds demonstrated superior or comparable binding scores to the reference compounds PKUMDL-LC-102, a GPX4 activator-type compound, and donepezil, an AChE inhibitor, suggesting their potential as viable computational hit candidates. However, docking scores alone cannot fully reflect ligand stability, interaction persistence, or the dynamic behavior of protein–ligand complexes. Therefore, the subsequent MD simulations were essential for distinguishing between initial binding affinity and sustained interaction behavior under simulated physiological conditions.

The persistent ligand–residue interactions played a particularly important role in maintaining NPACT00189’s stability in the allosteric pocket of GPX4. These interactions are consistent with the binding properties of known GPX4 activators, which generally exploit the electrostatic and hydrophobic nature of the allosteric site [20]. In this study, MD trajectories were analyzed to evaluate the structural stability and interaction persistence of the protein–ligand complexes. The results suggest that NPACT00189 maintained a relatively stable binding profile within the dynamic environments of both GPX4 and AChE during the simulation period. In contrast, although NPACT01210 was initially prioritized by docking and statistical ranking, MD simulations showed that it exhibited less persistent residence within the GPX4 allosteric pocket. This difference indicates that NPACT00189 may represent a more balanced dual-target binding candidate than NPACT01210.

The SiteMap analysis further supports this dual-target interpretation. Despite the volumetric difference between the AChE active-site gorge and the GPX4 allosteric pocket, both binding regions contain physicochemical features that may support ligand recognition. AChE possesses a deep and narrow active-site gorge that facilitates strong ligand interactions through multiple aromatic residues, whereas the GPX4 allosteric pocket represents a smaller and more flexible regulatory region [34,36,37]. The presence of hydrophobic/aromatic residues, hydrogen-bonding capacity, and acidic anchoring residues in both regions may provide a structural basis for the ability of certain plant-derived compounds to interact with both targets. However, such binding-pocket complementarity should not be overinterpreted as biological efficacy; it only supports the structural feasibility of dual binding.

The active-site gorge of AChE comprises two functionally distinct regions: the peripheral anionic site (PAS) at the entrance, characterized by the aromatic residue Trp286, and the acylation site (AS), which contains the catalytic triad Ser203, Glu334, and His447, as well as the critical Trp86 residue [34]. Effective AChE inhibitors typically interact with these regions, thereby blocking substrate access and preventing catalytic activity. In the present study, NPACT00189 showed persistent interaction behavior within the AChE active-site gorge, supporting a binding mode compatible with AChE inhibition. Nevertheless, this interpretation requires biochemical confirmation through direct AChE inhibition assays.

In the literature, Alameen et al. [20] conducted molecular docking and MD simulation studies on PKUMDL-LC-102, a known GPX4 activator-type compound, and elucidated its binding mechanism to GPX4. Their docking results indicated that the compound interacts predominantly with Asp23 and Met102. The MD simulations further revealed pronounced fluctuations in the ligand RMSD plot. The docking and MD simulation findings obtained in our study for PKUMDL-LC-102 are consistent with those reported in the literature. However, in the present work, PKUMDL-LC-102 was used mainly as a literature-based reference compound for workflow support and comparison, rather than as evidence of stable GPX4 binding under our simulation conditions.

In another study, Thai et al. [38] performed a screening of PubChem compounds to evaluate their binding potential against AChE and identified a candidate compound with favorable interactions within the active site. The reported compound formed key interactions with residues such as Asp74, Tyr124, and Tyr341, and subsequent MD simulations supported its potential as a promising AChE inhibitor. In the present study, NPACT00189 also maintained persistent contacts with residues located in or near the AChE active-site gorge, supporting its possible AChE-directed binding behavior. However, unlike experimentally tested AChE inhibitors, NPACT00189 remains a computationally proposed candidate, and its inhibitory activity must be confirmed experimentally.

Recent studies on ferroptosis-modulating compounds and multi-target AD-directed molecules support the rationale for exploring simultaneous modulation of cholinergic dysfunction and ferroptosis-related pathways [35,39,40,41]. Collectively, these reports underscore that effective intervention in complex conditions such as AD requires simultaneous modulation of multiple pathological mechanisms. These include established hallmarks such as cholinergic dysfunction as well as emerging cell death pathways such as ferroptosis. In this context, NPACT00189 represents a promising preliminary dual-target binding candidate. However, this interpretation should be considered preliminary and computational, as the present findings do not directly demonstrate biological efficacy or enzymatic GPX4 activation.

The therapeutic regulation of ferroptosis by small molecules has been investigated in different biological contexts. The successful identification of GPX4 inhibitors derived from mercaptosuccinic acid, as reported by Fatonah et al., supports the feasibility of targeting GPX4-related ferroptosis pathways through molecular docking-based approaches [42]. In another investigation exploring the effect of plant-derived compounds on ferroptosis, silibinin was found to attenuate ferroptosis in acute kidney injury by targeting FTH1 [43]. These findings highlight that the therapeutic regulation of ferroptosis by phytochemicals can occur through diverse molecular targets beyond GPX4, reinforcing the broader potential of natural products in modulating different aspects of this cell death pathway. However, since the present study focuses on AD-related dual targeting, these studies should be interpreted as supportive mechanistic examples rather than direct disease-specific validation.

In addition to structural stability, preliminary pharmacokinetic suitability of the lead compound NPACT00189 was evaluated using in silico ADMET prediction tools. Given the intended application in AD, BBB permeability is a critical parameter for evaluating the translational relevance of NPACT00189. The ADMET results indicated tool-dependent outcomes, with PreADMET suggesting potential brain exposure, whereas SwissADME and QikProp indicated limited passive BBB permeability. This discrepancy is understandable because different computational platforms rely on distinct physicochemical descriptors and training datasets. Importantly, NPACT00189 has a relatively high molecular weight and elevated topological polar surface area, which may restrict passive diffusion across the BBB. Physicochemical properties such as molecular weight, lipophilicity, hydrogen-bonding capacity, and polarity are important determinants of CNS drug-likeness and BBB penetration [44]. Therefore, despite its favorable in silico binding and MD stability profile, the actual brain availability of NPACT00189 remains uncertain. Experimental permeability and pharmacokinetic studies will be required to determine its CNS exposure.

Overall, the present findings suggest that NPACT00189 may act as a promising computational lead compound capable of engaging both GPX4 and AChE. Its interaction profile supports a possible AChE-directed inhibitory binding mode and a putative GPX4 allosteric modulatory interaction. However, these results should be interpreted with caution because they do not prove enzymatic inhibition, GPX4 activation, ferroptosis inhibition, or neuroprotective efficacy. The findings provide a structural and computational rationale for further experimental studies.

### 4.1. Limitations

While this study provides promising insights into the dual-target potential of plant-based compounds against GPX4 and AChE, it is subject to several limitations. First, all findings are based on in silico predictions; therefore, experimental validation through enzymatic and cellular assays is essential to confirm binding and biological activity. Second, although MD simulations and trajectory-based MM/GBSA calculations provide a more dynamic assessment than static docking alone, they cannot fully reproduce the complexity of cellular environments, protein regulation, metabolism, or pharmacokinetics. Third, the GPX4 findings should be interpreted as putative allosteric binding rather than proof of enzymatic activation. Finally, although preliminary ADMET predictions were performed, computational pharmacokinetic models may produce variable results depending on the prediction algorithms used. Consequently, experimental permeability and pharmacokinetic studies will be required to determine the actual brain availability of NPACT00189.

### 4.2. Future Directions

Building on the promising in silico findings, several key directions may further advance the translational potential of NPACT00189. First, the predicted dual-target interaction of NPACT00189 should be validated through biochemical assays, including AChE inhibition assays and GPX4 activity or stabilization assays, complemented by cell-based functional studies. Second, the compound’s ability to mitigate oxidative stress, lipid peroxidation, ferroptosis-related neuronal injury, and cholinergic dysfunction should be investigated in relevant neuronal or cellular models. Third, BBB permeability and pharmacokinetic studies will be necessary to clarify its CNS availability. Fourth, medicinal chemistry optimization may be required to improve CNS drug-likeness, metabolic stability, and brain exposure while preserving the dual-target interaction profile. Finally, preclinical evaluation in suitable animal models of neurodegeneration will be necessary to determine therapeutic efficacy and safety. Collectively, these steps will be important for translating computational predictions into viable therapeutic strategies for neurodegenerative disorders such as AD.

## 5. Conclusions

In this molecular modeling and dynamics study, a comprehensive virtual screening of plant-based compounds identified NPACT00189 as promising dual-target hit compound against Alzheimer’s disease by simultaneously targeting GPX4 and AChE. Docking analysis indicated high binding affinity towards both target proteins, exceeding that of the reference GPX4 activator PKUMDL-LC-102. Molecular Dynamics simulations suggested that NPACT00189 exhibited stable binding in both the AChE active gorge and the GPX4 allosteric site, maintaining key interactions throughout the 250 ns trajectory, thus supporting its potential as a balanced dual-target modulator. Conversely, while NPACT01210 showed good stability within the AChE complex, its significant displacement from the GPX4 allosteric pocket after 47 ns suggests that its activity may be predominantly restricted to AChE inhibition. These results provide computational evidence that NPACT00189 may represent a promising preliminary lead candidate for further evaluation in AD-related drug discovery. However, the promising dual-target profile of NPACT00189 must be rigorously validated through subsequent in vitro enzyme inhibition assays and in vivo studies to confirm its efficacy, bioavailability, and therapeutic potential.

## Figures and Tables

**Table 1:** Docking scores and MM/GBSA binding affinities (kcal/mol) of selected compounds against GPX4 and AChE targets.

Compounds	GPX4 Docking	GPX4 MM/GBSA	AChE Docking	AChE MM/GBSA	Z_GPX4_	Z_AChE_	C_score_
NPACT00189	−6.720	−45.06	−8.983	−66.68	3.6777	0.6734	4.3510
NPACT01210	−5.813	−39.89	−9.640	−92.67	1.5956	1.7828	3.3783
Donepezil	-	-	−9.456	−90.74	-	-	-
PKUMDL-LC-102	−5.671	−18.05	-	-	-	-	-

**Table 2:** Chemical structures and molecular descriptors of the top dual-target compounds.

Compound ID	NPACT00189
**Structure**	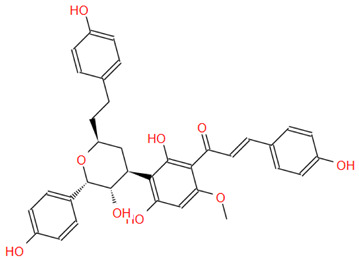
**ZINC ID**	ZINC000095098974
**Molecular Formula**	C35H34O9
**Molecular Weight (g/mol)**	598.648
**Canonical SMILES**	COc1cc(O)c([C@@H]2C[C@H](CCc3ccc(O)cc3)O[C@@H](c3ccc(O)cc3)[C@H]2O)c(O)c1C(=O)/C=C/c1ccc(O)cc1
**Compound ID**	**NPACT01210**
**Structure**	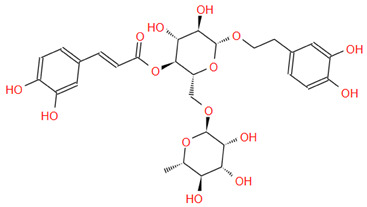
**ZINC ID**	ZINC000008234345
**Molecular Formula**	C29H36O15
**Molecular Weight (g/mol)**	624.592
**Canonical SMILES**	C[C@@H]1O[C@@H](OC[C@H]2O[C@@H](OCCc3ccc(O)c(O)c3)[C@H](O)[C@@H](O)[C@@H]2OC(=O)/C=C/c2ccc(O)c(O)c2)[C@H](O)[C@H](O)[C@H]1O

**Table 3:** Mean trajectory-based MM/GBSA binding free energies of selected ligand–protein complexes.

Complex	Mean ± SD ΔG Bind (kcal/mol)
NPACT00189–AChE	−46.97 ± 13.65
NPACT00189–GPX4	−36.92 ± 7.96
NPACT01210–AChE	−73.90 ± 10.28
NPACT01210–GPX4	−37.83 ± 9.76

**Table 4:** Binding pocket complementarity analysis of the selected Site 1 pockets for GPX4 and AChE.

Property	AChE (7D9Q)	GPX4 (7U4I)	Interpretation
Volume (Å^3^)	454.5	91.6	Size difference
Don/acc ratio	0.819	0.991	Balanced H-bond capacity
Philic score	0.853	0.629	Polar complementarity
Phobic score	1.097	0.283	Hydrophobic character
Key aromatic residues	Trp86, Phe295, Tyr341, Trp439	Phe100, Met102	Conserved aromatic recognition
Acidic anchors	Asp74, Glu292	Asp21, Asp23	Electrostatic motifs

## Data Availability

The data used in this study were obtained by downloading the NPACT, HIT, and HIM collections available within the COCONUT (Collection Of Open Natural Products) database (https://coconut.naturalproducts.net/ (accessed on 1 November 2025)). All compound sets were retrieved in standardized formats and locally processed for virtual screening.

## Supplementary Materials

The following supporting information can be downloaded at: https://www.mdpi.com/article/10.3390/pharmaceutics18070798/s1, Table S1. Phytochemical compounds ranked by composite Z-scores and C-scores indicating dual binding potential against GPX4 and AChE based on molecular docking analyses; Table S2. Statistical parameters used for Z-score normalization; Table S3. SiteMap analysis of the selected Site 1 binding pocket properties for GPX4 and AChE; Figure S1. Redocking verification for H1R in the 7D9Q complex: superposition of the crystal initial pose and the post-redocking pose (evaluation by RMSD); Figure S2. Timeline of interactions of NPACT00189 in GPX4 during 250 ns MD simulation; Figure S3. Timeline of interactions of NPACT01210 in GPX4 during 250 ns MD simulation; Figure S4. 250 ns MD simulation analysis of PKUMDL-LC-102 within the allosteric site of GPX4, illustrating the timeline of interactions profiles; Figure S5. Timeline of interactions of NPACT00189 in AChE during 250 ns MD simulation; Figure S6. Timeline of interactions of NPACT01210 in AChE during 250 ns MD simulation; Figure S7. 250 ns MD simulation analysis of Donepezil within the active gorge of AChE, illustrating the timeline of interactions profiles; Figure S8. Time-dependent hydrogen bond evolution between selected ligands and their target proteins during 250 ns MD simulations; Figure S9. Trajectory-based MM/GBSA binding free energy profiles of NPACT01210 complexes.

## Abbreviations

The following abbreviations are used in this manuscript:

AD	Alzheimer’s disease
ACh	Acetylcholine
AChE	Acetylcholinesterase
ChEIs	Cholinesterase inhibitors
COCONUT	COlleCtion of Open Natural prodUcTs
GPX4	Glutathione peroxidase 4
HIM	Herbal Ingredients In Vivo Metabolism Database
HIT	Herbal Ingredients’ Targets Database
MD	Molecular Dynamics
MM/GBSA	Molecular Mechanics/Generalized Born Surface Area
NPACT	Naturally Occurring Plant-based Anti-cancer Compound–Activity–Target Database
RMSD	Root-Mean-Square Deviation
RMSF	Root-Mean-Square Fluctuation
SAR	Structure-activity relationship
