# Supplementary material for: Dual Targeting of AChE Inhibition and GPX4 Binding by Plant-Derived Compounds for the Treatment of Alzheimer’s Disease: Insights from Molecular Docking and Molecular Dynamics Simulations
Source: Pharmaceutics. 2026 Jun 29;18(7):798. doi: 10.3390/pharmaceutics18070798 (PMC13414810; doi:10.3390/pharmaceutics18070798)
Supplement: Supplementary file 1 [file pharmaceutics-18-00798-s001.zip › pharmaceutics-4363480-supplementary.pdf]

Dual Targeting of AChE Inhibition and GPX4 Binding by Plant-Derived Compounds for the Treatment of Alzheimer's Disease: Insights from Molecular Docking and Molecular Dynamics Simulations

Suheda Rumeysa Osmanlıoğlu Dağ, Mehmet Abdullah Alagöz

**Contents**	
**Table S1.** Phytochemical compounds ranked by composite Z-scores and C-scores indicating dual binding potential against GPX4 and AChE based on molecular docking analyses	**2**
**Table S2.** Statistical Parameters Used for Z-score Normalization	**3**
**Table S3.** SiteMap analysis of the selected Site 1 binding pocket properties for GPX4 and AChE.	**3**
**Figure S1.** Redocking verification for H1R in the 7D9Q complex: superposition of the crystal initial pose and the post-redocking pose (evaluation by RMSD)	**4**
**Figure S2.** Timeline of interactions of NPACT00189 in GPX4 during 250 ns MD simulation	**5**
**Figure S3.** Timeline of interactions of NPACT01210 in GPX4 during 250 ns MD simulation	**6**
**Figure S4.** 250 ns MD simulation analysis of PKUMDL-LC-102 within the allosteric site of GPX4, illustrating the timeline of interactions profiles	**7**
**Figure S5.** Timeline of interactions of NPACT00189 in AChE during 250 ns MD simulation	**8**
**Figure S6.** Timeline of interactions of NPACT01210 in AChE during 250 ns MD simulation	**9**
**Figure S7.** 250 ns MD simulation analysis of Donepezil within the active gorge of AChE, illustrating the timeline of interactions profiles	**10**
**Figure S8.** Time-dependent hydrogen bond evolution between selected ligands and their target proteins during 250 ns MD simulations.	**11**
**Figure S9.** Trajectory-based MM/GBSA binding free energy profiles of NPACT01210 complexes.	**12**

Table S1. Phytochemical compounds ranked by composite Z-scores and C-scores indicating dual binding potential against GPX4 and AChE based on molecular docking analyses (kcal/mol)

No	Compound	Gpx4	Ache	Zgpx4	Zache	Cscore
1.	NPACT00189	-6,72	-8,98	-3,678	-0,673	4,351
2.	NPACT01210	-5,81	-9,64	-1,596	-1,783	3,378
3.	Compound 913	-5,78	-9,54	-1,527	-1,615	3,142
4.	NPACT00845	-6.00	-9.00	-2,03	-0,707	2,737
5.	NPACT00327	-4,78	-10,6	0,761	-3,396	2,635
6.	Compound 290	-5,78	-8,95	-1,527	-0,623	2,15
7.	NPACT00544	-5,53	-9,18	-0,955	-1,01	1,964
8.	Compound 73	-5,01	-9,64	0,235	-1,783	1,548
9.	NPACT00378	-5,26	-9,19	-0,337	-1,026	1,363
10.	Compound 677	-5,27	-9,11	-0,36	-0,892	1,252
11.	Compound 562	-5,92	-8,21	-1,847	0,621	1,226
12.	Compound1070	-5,92	-8,21	-1,847	0,621	1,226
13.	NPACT00918	-4,95	-9,43	0,372	-1,43	1,058
14.	NPACT00995	-4,61	-9,84	1,15	-2,119	0,969
15.	NPACT00809	-5,1	-9,1	0,029	-0,875	0,846
16.	Compound 802	-5,72	-8,21	-1,39	0,621	0,769
17.	NPACT01553	-5,6	-8,28	-1,115	0,503	0,612
18.	NPACT01194	-5,73	-8,07	-1,413	0,856	0,556
19.	NPACT00800	-4,84	-9,23	0,624	-1,094	0,47
20.	NPACT00341	-4,79	-9,25	0,738	-1,127	0,389
21.	NPACT00384	-5,41	-8,39	-0,68	0,318	0,362
22.	Compound 314	-5,25	-8,6	-0,314	-0,035	0,349
23.	NPACT00381	-5,48	-8,28	-0,841	0,503	0,337
24.	NPACT00212	-4,74	-9,26	0,853	-1,144	0,291
25.	NPACT00944	-4,5	-9,54	1,402	-1,615	0,213
26.	NPACT00808	-5,38	-8,3	-0,612	0,47	0,142
27.	NPACT00680	-4,9	-8,95	0,487	-0,623	0,136
28.	NPACT00923	-4,92	-8,9	0,441	-0,539	0,098
29.	Compound 446	-5,12	-8,62	-0,017	-0,068	0,085
30.	Compound 1092	-4,82	-8,93	0,67	-0,589	-0,08
31.	Compound 833	-4,95	-8,73	0,372	-0,253	-0,119
32.	NPACT00757	-4,97	-8,7	0,326	-0,203	-0,124
33.	NPACT00660	-4,8	-8,93	0,715	-0,589	-0,126
34.	NPACT00553	-4,96	-8,66	0,349	-0,135	-0,214
35.	NPACT01533	-5,68	-7,67	-1,298	1,529	-0,231
36.	NPACT00878	-5,23	-8,28	-0,269	0,503	-0,235

37.	NPACT00272	-4,5	-9,26	1,402	-1,144	-0,258
38.	Compound 532	-5,05	-8,48	0,143	0,167	-0,31
39.	Compound 162	-5,14	-8,31	-0,063	0,453	-0,39
40.	NPACT00930	-5,3	-8,09	-0,429	0,823	-0,394
41.	NPACT01554	-5,45	-7,87	-0,772	1,192	-0,421
42.	Compound 413	-5,06	-8,39	0,12	0,318	-0,439
43.	NPACT00031	-5,17	-8,19	-0,131	0,655	-0,523
44.	Compound 1105	-4,88	-8,56	0,532	0,033	-0,565
45.	NPACT00083	-5,24	-8,05	-0,291	0,89	-0,599
46.	NPACT00669	-5,41	-7,76	-0,68	1,377	-0,697
47.	NPACT00540	-4,97	-8,34	0,326	0,402	-0,729
48.	NPACT00511	-4,82	-8,54	0,67	0,066	-0,736
49.	NPACT00262	-4,95	-8,36	0,372	0,369	-0,741
50.	NPACT00543	-4,61	-8,82	1,15	-0,404	-0,746
51.	Compound 875	-5,37	-7,76	-0,589	1,377	-0,789
52.	NPACT00708	-5,22	-7,91	-0,246	1,125	-0,88
53.	NPACT00807	-4,57	-8,79	1,242	-0,354	-0,888
54.	NPACT00035	-4,87	-8,31	0,555	0,453	-1,008
55.	Compound 495	-4,72	-8,5	0,898	0,133	-1,032
56.	NPACT00542	-4,98	-8,08	0,304	0,839	-1,143
57.	NPACT00742	-4,89	-8,19	0,509	0,655	-1,164
58.	Compound 110	-5,06	-7,89	0,12	1,159	-1,279
59.	NPACT01309	-4,7	-8,3	0,944	0,47	-1,414
60.	NPACT00981	-4,71	-8,28	0,921	0,503	-1,425
61.	NPACT00376	-4,78	-8,13	0,761	0,755	-1,517
62.	NPACT00949	-5,01	-7,8	0,235	1,31	-1,545
63.	NPACT00383	-4,75	-8,04	0,83	0,907	-1,736
64.	NPACT00545	-4,79	-7,96	0,738	1,041	-1,779
65.	Compound 1098	-4,8	-7,85	0,715	1,226	-1,941
66.	NPACT00677	-4,55	-8,16	1,287	0,705	-1,992
67.	NPACT00088	-4,57	-8,07	1,242	0,856	-2,098
68.	NPACT00799	-4,54	-7,96	1,31	1,041	-2,351

Table S2. Statistical Parameters Used for Z-score Normalization

**Parameter**	**GPX4 Docking Scores**	**AChE Docking Scores**
**Mean (**μ**)**	-5.113 kcal/mol	-8.579 kcal/mol
**Standard Deviation (**σ**)**	0.437 kcal/mol	0.595 kcal/mol

Table S3. SiteMap Analysis of Binding Pocket Properties for GPX4 and AChE

**Property**	**AChE (7D9Q) Active-Site Gorge**	**GPX4 (7U4I) Allosteric Pocket**

**Site Score**	1.147	0.639
**Dscore**	1.180	0.620
**Size**	188	26
**Balance**	1.286	0.450
**Contact**	1.105	0.708
**Don/acc**	0.819	0.991
**Enclosure**	0.864	0.576
**Exposure**	0.449	0.816
**Philic**	0.853	0.629
**Phobic**	1.097	0.283
**Volume**	454.475	91.581
**Residues**	Gln71, Tyr72, Val73, Asp74, Thr83, Trp86, Asn87, Pro88, Gly120, Gly121, Gly122, Tyr124, Ser125, Gly126, Leu130, Tyr133, Gly202, Ser203, Trp286, Leu289, Glu292, Ser293, Val294, Phe295, Tyr337, Phe338, Tyr341, Gly342, Val365, Trp439, His447, Gly448	Asp21, Ile22, Asp23, Lys90, Ala93, Ala94, Asn97, Val98, Lys99, Phe100, Asp101, Met102

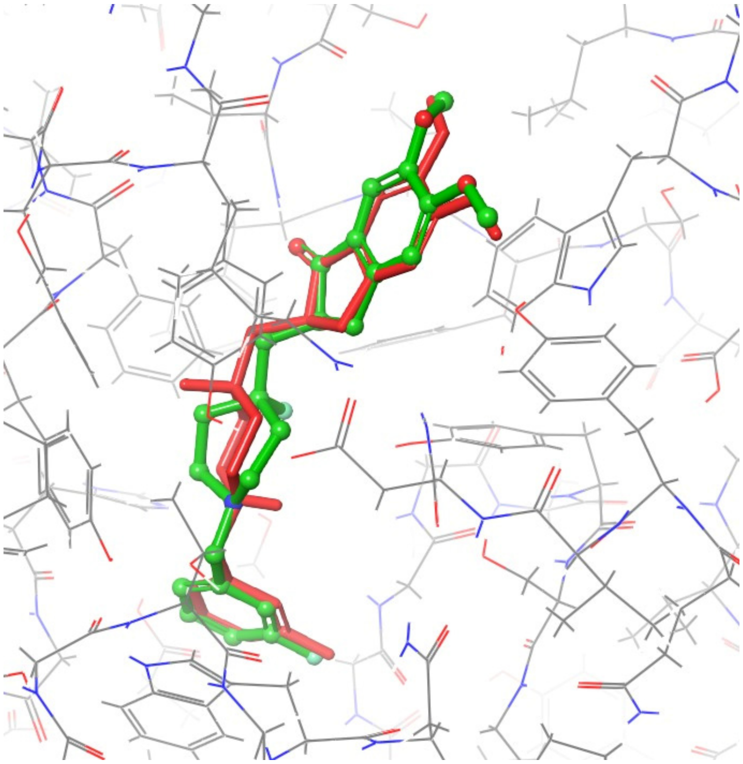

Figure S1. Redocking verification for H1R in the 7D9Q complex: superposition of the crystal initial pose and the post-redocking pose (evaluation by RMSD).

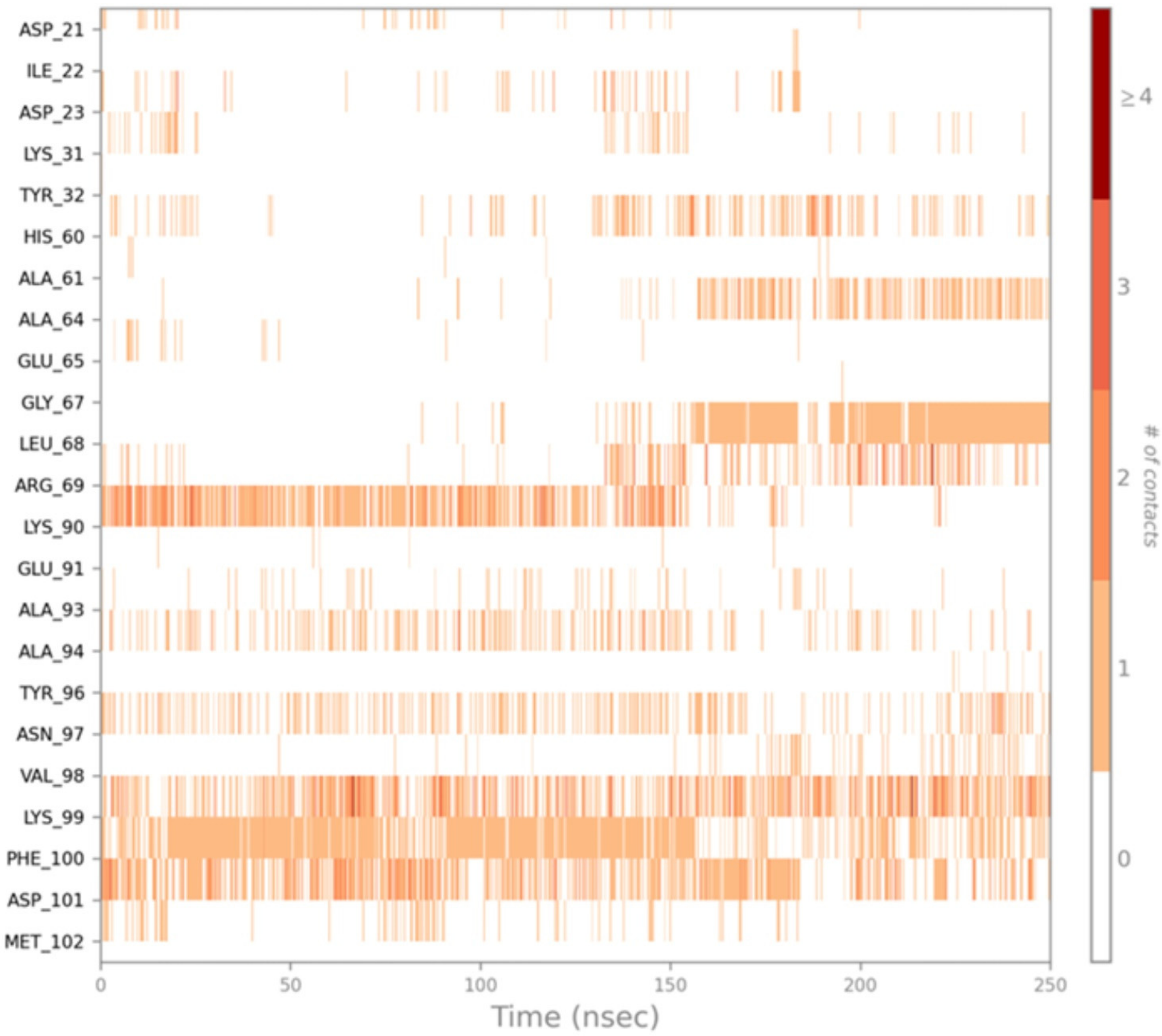

Figure S2. Timeline of interactions of NPACT00189 in GPX4 during 250 ns MD simulation.

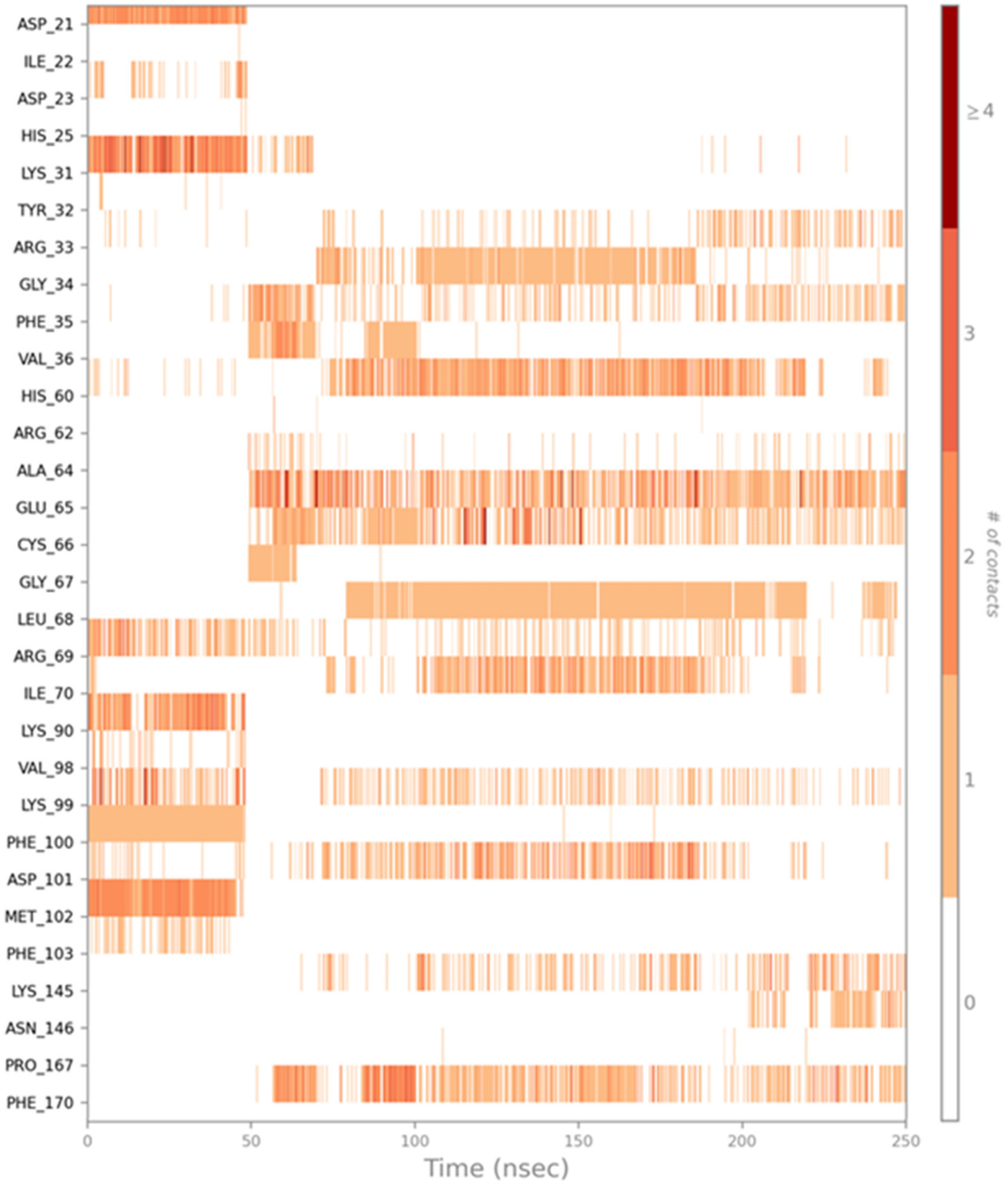

Figure S3. Timeline of interactions of NPACT01210 in GPX4 during 250 ns MD simulation.

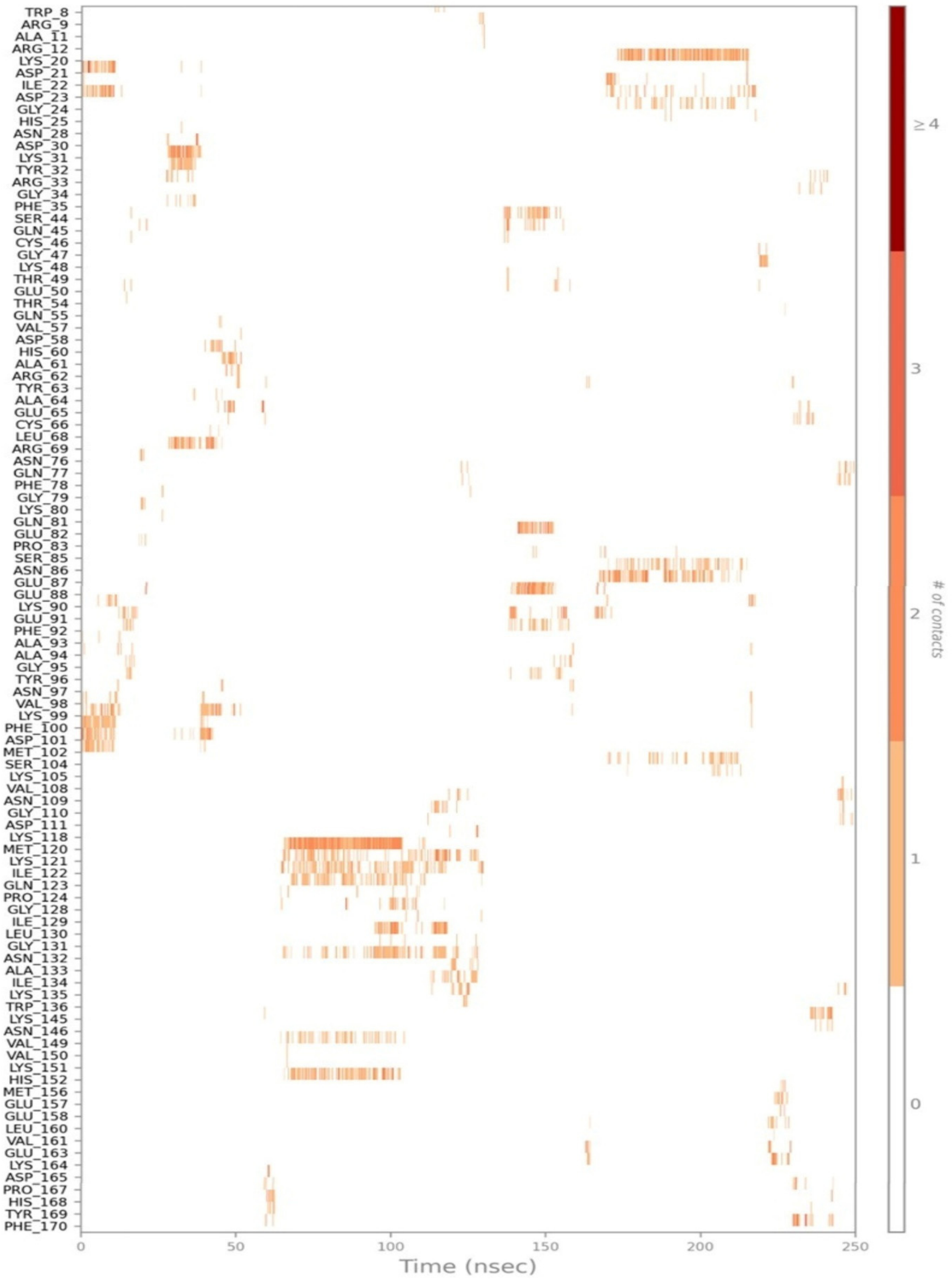

Figure S4. 250 ns MD simulation analysis of PKUMDL-LC-102 within the allosteric site of GPX4, illustrating the timeline of interactions profiles.

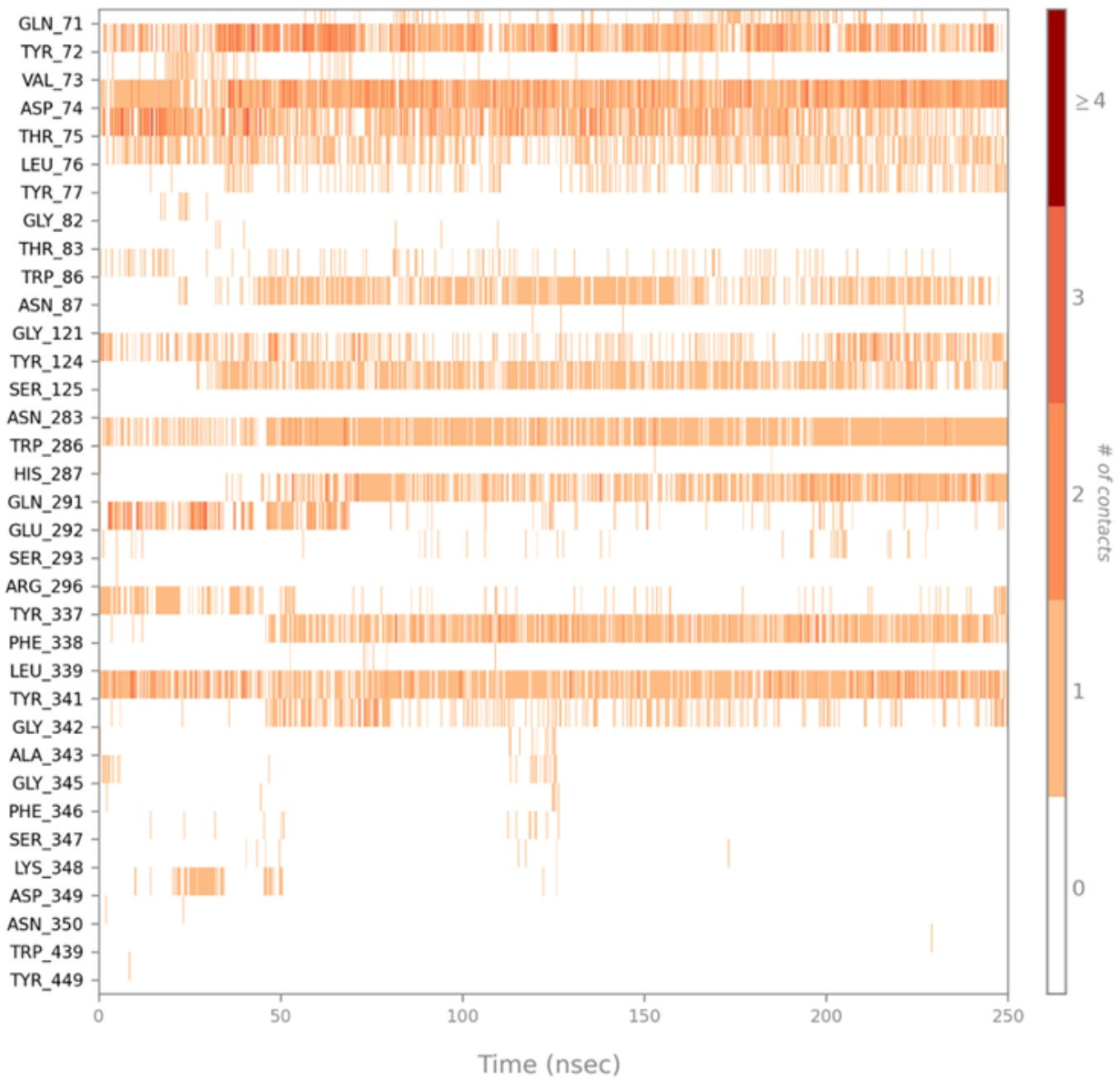

Figure S5. Timeline of interactions of NPACT00189 in AChE during 250 ns MD simulation.

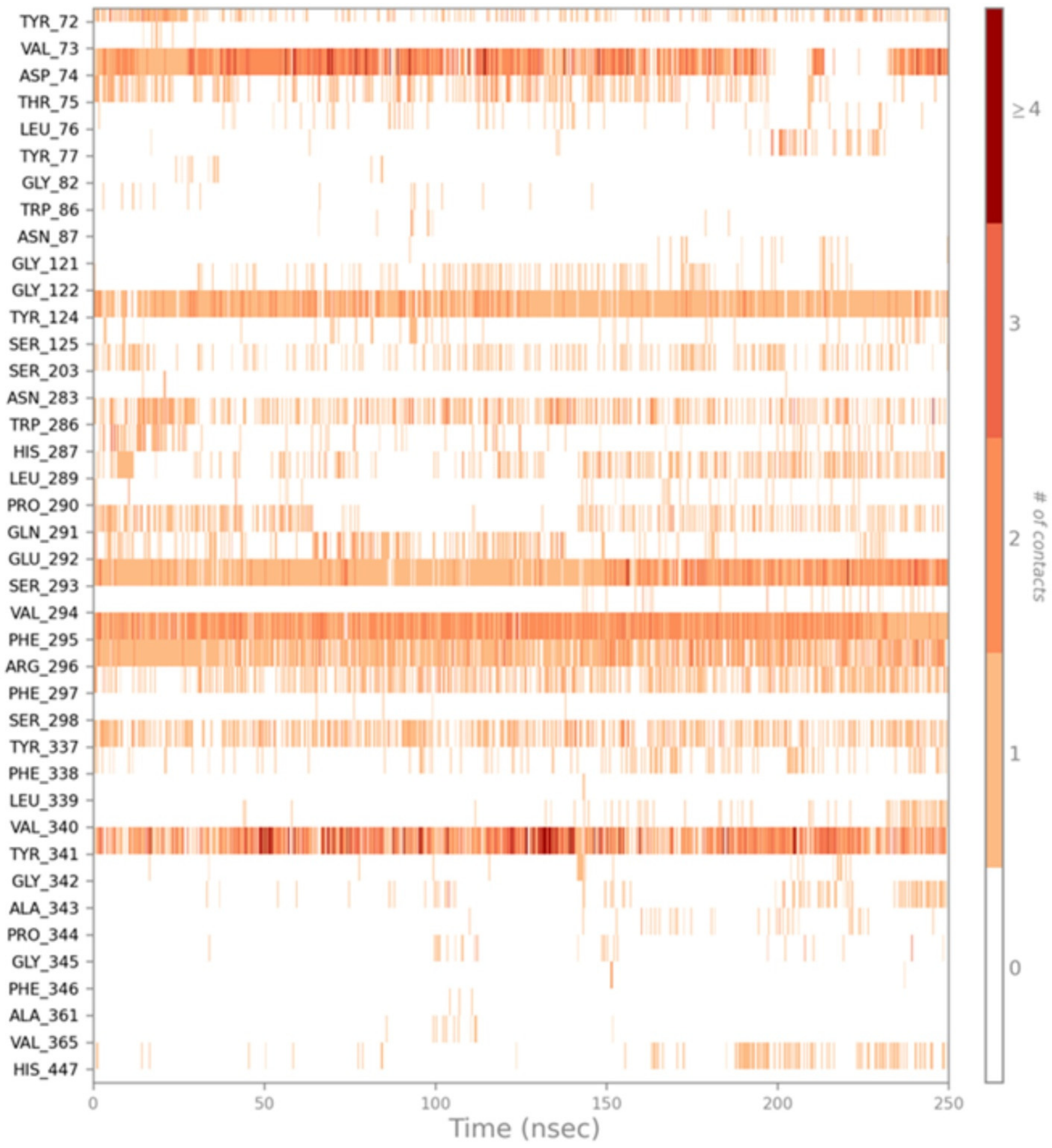

Figure S6. Timeline of interactions of NPACT01210 in AChE during 250 ns MD simulation**.**

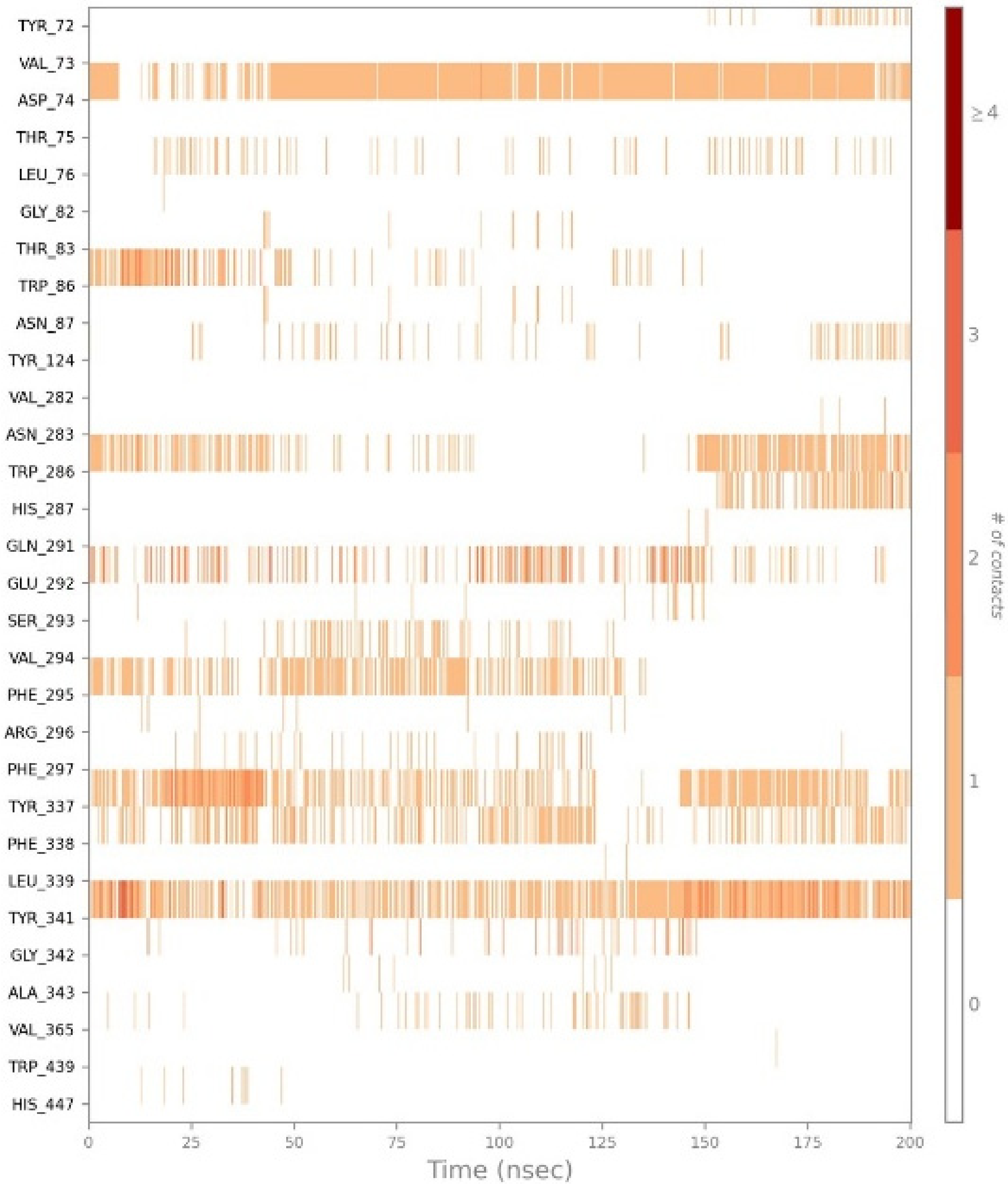

Figure S7. 250 ns MD simulation analysis of Donepezil within the active gorge of AChE, illustrating the timeline of interactions profiles.

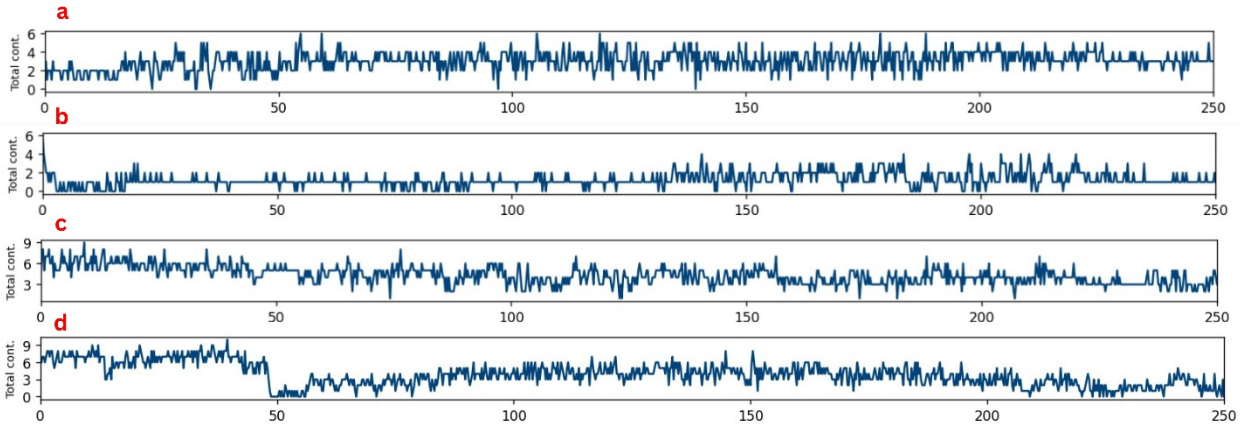

Figure S8. Time-dependent hydrogen bond evolution between selected ligands and their target proteins during 250 ns MD simulations.

The line plots show the number of hydrogen bonds formed between the ligand and protein throughout the 250 ns production MD trajectories. (a) NPACT00189–AChE, (b) NPACT00189–GPX4, (c) NPACT01210–AChE, and (d) NPACT01210–GPX4. The X-axis represents simulation time in nanoseconds, and the Y-axis represents the total number of hydrogen bonds. The dynamic H-bond profiles provide additional support for the temporal persistence and fluctuation of ligand–protein interactions during the simulations.

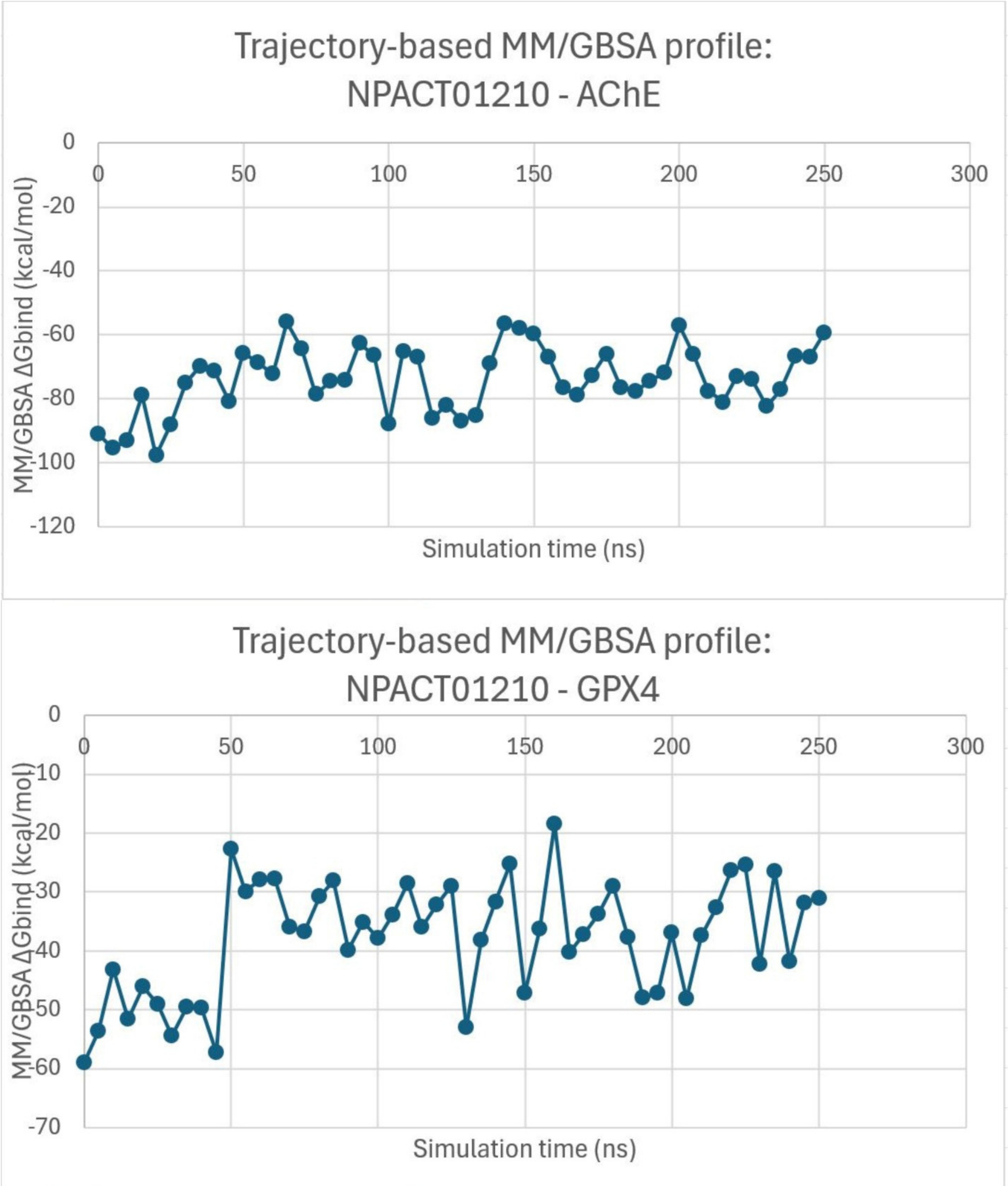

Figure S9. Trajectory-based MM/GBSA binding free energy profiles of NPACT01210 complexes.
